# Modulation of anti-cardiac fibrosis immune responses by changing M2 macrophages into M1 macrophages

**DOI:** 10.1186/s10020-024-00858-z

**Published:** 2024-06-15

**Authors:** Shiqi Chen, Kan Wang, Zhengfeng Fan, Tingwen Zhou, Rui Li, Bingxia Zhang, Jie Chen, Jiangyang Chi, Keke Wei, Jincheng Liu, Zongtao Liu, Jingwei Ma, Nianguo Dong, Junwei Liu

**Affiliations:** 1 Department of Cardiovascular Surgery, Union Hospital, Tongji Medical College, Huazhong University of Science and Technology, Wuhan, 430022 China; 2 Department of Immunology, Tongji Medical College, Huazhong University of Science & Technology, Wuhan, 430030 China; 3 Department of Biochemistry & Molecular Biology, Tongji Medical College, Huazhong University of Science & Technology, Wuhan, 430030 China; 4 Key Laboratory of Organ Transplantation, Ministry of Education, NHC Key Laboratory of Organ Transplantation, Key Laboratory of Organ Transplantation, Chinese Academy of Medical Sciences, Wuhan, China

**Keywords:** Cardiac fibrosis, Macrophage, Glycogen, TMEM175

## Abstract

**Background:**

Macrophages play a crucial role in the development of cardiac fibrosis (CF). Although our previous studies have shown that glycogen metabolism plays an important role in macrophage inflammatory phenotype, the role and mechanism of modifying macrophage phenotype by regulating glycogen metabolism and thereby improving CF have not been reported.

**Methods:**

Here, we took glycogen synthetase kinase 3β (GSK3β) as the target and used its inhibitor NaW to enhance macrophage glycogen metabolism, transform M2 phenotype into anti-fibrotic M1 phenotype, inhibit fibroblast activation into myofibroblasts, and ultimately achieve the purpose of CF treatment.

**Results:**

NaW increases the pH of macrophage lysosome through transmembrane protein 175 (TMEM175) and caused the release of Ca^2+^ through the lysosomal Ca^2+^ channel mucolipin-2 (Mcoln2). At the same time, the released Ca^2+^ activates TFEB, which promotes glucose uptake by M2 and further enhances glycogen metabolism. NaW transforms the M2 phenotype into the anti-fibrotic M1 phenotype, inhibits fibroblasts from activating myofibroblasts, and ultimately achieves the purpose of treating CF.

**Conclusion:**

Our data indicate the possibility of modifying macrophage phenotype by regulating macrophage glycogen metabolism, suggesting a potential macrophage-based immunotherapy against CF.

**Graphical Abstract:**

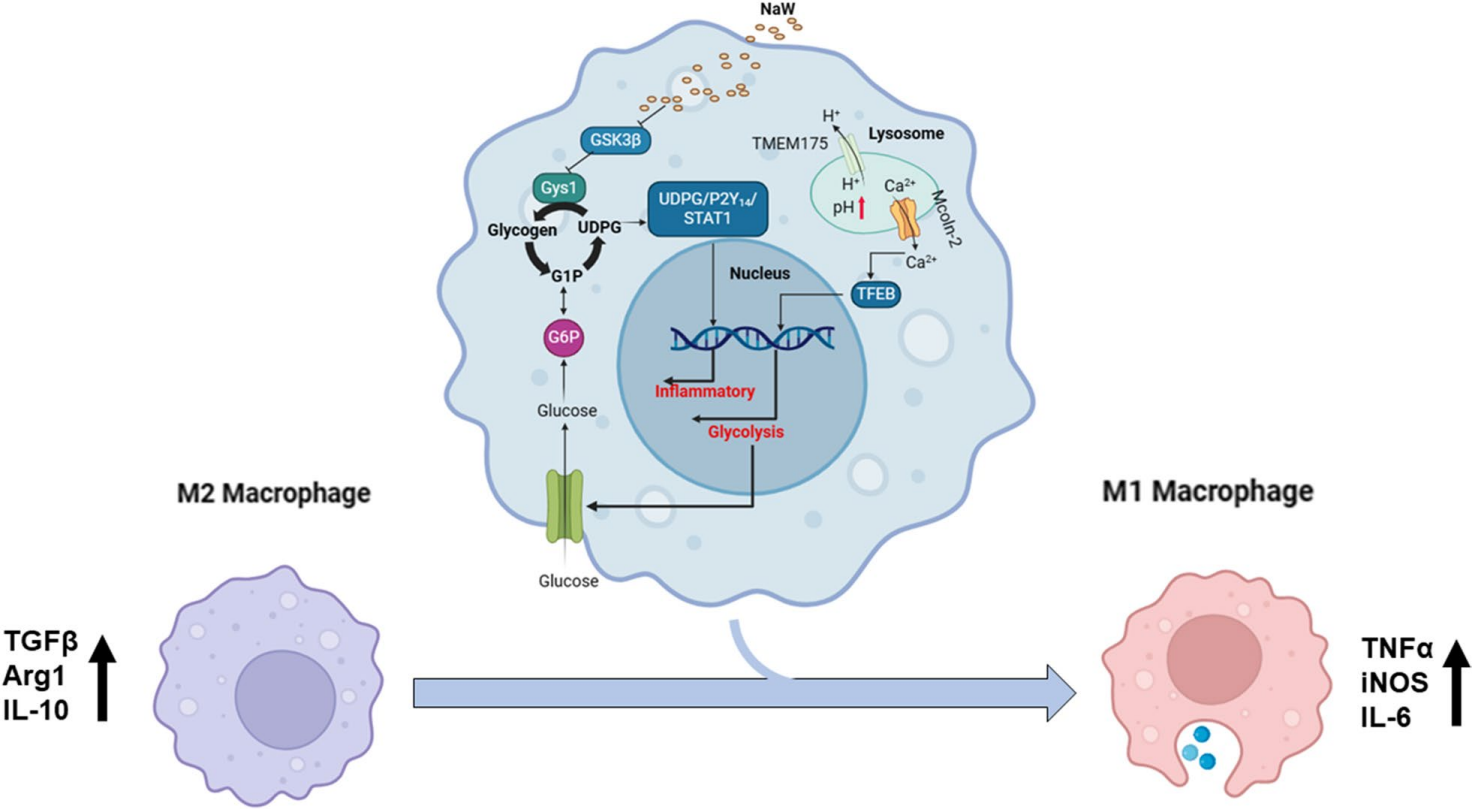

**Supplementary Information:**

The online version contains supplementary material available at 10.1186/s10020-024-00858-z.

## Introduction

Cardiovascular diseases cause approximately 31% of deaths worldwide (Murtha et al. 2017), and cardiac fibrosis (CF) is caused by excessive deposition of collagen fibers in myocardial tissue structures and is a common pathological alteration observed in the end stage of various cardiovascular disease (Fang et al. 2017). Epidemiological studies have shown that CF is an independent risk factor for cardiovascular diseases and greatly increases the morbidity and mortality of cardiovascular diseases such as myocardial infarction (MI) and cardiac hypertrophy (Beltrami et al. 1994). The prevention and attenuation of CF are important for preventing the occurrence and development of various cardiovascular diseases.

After initial injury, cardiac fibroblasts are activated and subsequently differentiate into myofibroblasts, which are the major mediators of pathological remodeling. Myofibroblasts exhibit proliferative and secretory properties and contribute to the turnover of the extracellular matrix (ECM) and collagen deposition. Continuous myofibroblasts action leads to fibrotic scarring and cardiac dysfunction (Jellis et al. 2010). Prescribed therapeutic agents for the treatment of cardiovascular diseases, such as angiotensin II (Ang II) inhibitors, can alleviate CF. New therapeutic strategies for CF have also shown certain levels of efficacy; for example, engineered chimeric antigen receptor-T cells (CAR-T) have been used to target myofibroblasts (Aghajanian et al. 2019), and single-cell ribonucleic acid (RNA) sequencing (RNA-seq) has been used to verify a new therapeutic approach to fibrosis by inducing stem cells to differentiate into quiescent fibroblasts (Zhang et al. 2019). However, its clinical application has been limited due to side effects and limited efficacy (Liu et al. 2021). Therefore, further exploration into new intervention targets and drugs that can reverse or delay the progression of CF is needed.

Although activated myofibroblasts are the main effector cells in fibrotic hearts, other cell types and cytokines may also be involved in regulating the fibrotic response, and among the contributing cells, macrophages play a key regulatory role (Wynn and Barron 2010). Macrophages show remarkable plasticity, and monocytes/macrophages can differentiate into M1 (classically activated) or M2 (alternately activated) macrophage subclasses under different microenvironmental conditions. M1 macrophages secrete tumor necrosis factor-α (TNF-α) and nitric oxide (NO) and release matrix metalloproteinases (MMPs), degrading the extracellular matrix, which is involved in initiating inflammation after tissue injury. In contrast, M2 macrophages release IL-10 and transforming growth factor (TGF)-β, which are involved in tissue repair and healing and are key cells in the formation of CF (Rurik et al. 2021; Vannella and Wynn 2017; Gordon 2003). Finding suitable targets to regulate the phenotype acquisition and functional changes of macrophages is expected to be a potential strategy for CF treatment.

As professional phagocytes, macrophages exhibit a clear ability to take up many extracellular substances and effectively degrade them in lysosomes. This degradation process strictly depends on the pH of acidic lysosomes (Casey et al. 2010; Appelqvist et al. 2013). Our previous study showed that increasing lysosomal pH resulted in the release of calcium from lysosomes, which led to the activation of transcription factor EB (TFEB), a cytoplasmic transcription factor that is a key regulator of lysosomal biogenesis and glucose metabolism (Chen et al. 2018). In another report, we showed that M1 macrophages can take up a large amount of glucose, which is consumed in glycogen synthesis, to promote glycogen metabolism and activate the UDPG-P2Y_14_ signaling pathway, promoting inflammatory macrophage gene expression (Ma et al. 2020). Therefore, increasing the lysosomal pH and enhancing glycogen metabolism are closely related to the transformation of M2 macrophages into M1 macrophages, which is necessary to alleviate or reverse CF. Sodium tungstate (NaW), a weakly basic small-molecule drug, is a phosphatase inhibitor that phosphorylates the GSK-3β regulatory subunit at serine 9 and reduces the catalytic activity of this type of enzyme. To date, NaW has mainly been evaluated and shown to be an effective and safe hypoglycemia treatment agent in the context of diabetes in preclinical studies. However, the effects of NaW on macrophages have not been explored. In this study, we provide evidence showing that enhancing M2 macrophage glycogen metabolism in CF cells drives the re-acquisition of an M1 phenotype, antagonizing CF pathways. Moreover, NaW, an immunomodulator, promoted M2 macrophage glucose uptake by regulating the lysosomal pH, providing material needed for glycogen synthesis in macrophages.

## Materials and methods

### Data processing

We downloaded one heart failure (HF) RNA-seq dataset (GSE76701) and one HF single-cell RNA-seq dataset (GSE161740) from the Gene Expression Omnibus (GEO) database. After standardization, the samples without grouping information were excluded, and ultimately, 5 samples were obtained from the GSE161740 dataset, and 4 paired tissue samples were obtained from the GSE76701 dataset.

### Single-cell quality control and dimension reduction clustering

We selected cells with more than 300 expressed genes, fewer than 5500 expressed genes in total. Among these genes, with fewer than 10% of the candidates were mitochondrial genes, and less than 3% were red blood cell genes. A total of 15,749 cells were retained for analysis. Then, 3000 hypervariable genes were selected for analysis, and the number of principal components (PCs) was set to 15 for cell clustering. These clusters were displayed in the form of a t-distributed stochastic neighbor embedding (t-SNE) diagram. First, we identified the 10 markers that were the most significantly differently expressed (Supplementary file 1) in each cluster using the “FindAllmarkers” function. After identifying a group of macrophages, we isolated the macrophages and ultimately obtained 11 macrophage subclusters. The identified of the ten markers most closely associated with a macrophage subtypes were confirmed (Supplementary file 2).

#### Human samples

The Clinical Trials Ethics Committee of the Huazhong University of Science and Technology approved a clinical trial. All the samples were recruited from the Union Hospital affiliated with Tongji Medical College of Huazhong University of Science and Technology. Fibrotic left ventricular myocardium (FLVM) samples were obtained from end-stage HF patients at the time of heart transplantation. Control normal left ventricular myocardium (NLVM) samples were obtained from the left ventricular myocardium of nonfailing unused donor hearts. Written informed consent was obtained from all patients.

#### Animals

Male wild-type C57BL/6J mice and 8-week-old nude mice were purchased from Vital River Laboratory Animal Technology Co., Ltd. (Beijing, China). For the study, all mice were maintained under specific-pathogen-free (SPF) conditions at the Animal Care and Use Committee of Tongji Medical College. Euthanasia of the animals was performed in a carbon dioxide chamber followed by cervical dislocation.

#### MI induction and study design

Male C57BL/6J wild-type (WT) littermate mice (8 weeks) were used to establish models of MI, which was induced as described previously (Gao et al. 2010). Briefly, mice were exposed to a 5% isoflurane inhaler at an oxygen flow rate of 1 L/min. The mice were then continuously monitored throughout the procedure, and complete anesthesia was confirmed by the lack of pedal reflexes. After the left chest was incised, the pectoralis major and pectoralis minor were directly incised. The fourth intercostal space was gently opened with a clamp, and the heart was gently squeezed. The left anterior descending coronary artery was ligated with 6–0 silk thread. Immediately after the ligation, the heart was placed back into the proper position, the chest was closed, and air was expelled. NaW was administered at a concentration of 100 mg kg^−1^ from days 11 to 38 after MI. To determine cardiac function, echocardiograms were performed on days 1 and 38 after MI induction using a Vevo 2100 high-resolution microimaging system. The scarred area was evaluated using Masson’s trichrome method and calculated as the ratio of fibrotic tissue area to the total left ventricular tissue area.

#### Immunofluorescence staining

Briefly, after being dried at room temperature for 15 min, frozen sections of aortic valves were fixed in 4% paraformaldehyde (PFA) for 20 min and then permeabilized with 0.1% Triton X-100 in phosphate-buffered saline (PBS) for another 20 min. Next, the tissues were incubated with primary antibody, followed by incubation with fluorescently conjugated secondary antibody (Abcam) and counterstaining with 4′,6‐diamidino‐2‐phenylindole (DAPI).

#### Cell isolation and stimulation

Macrophages were isolated from the infarcted LV tissues on day 38 post-MI, and cardiac fibroblasts were isolated from control (noninfarcted) LV tissues. Macrophages and cardiac fibroblasts were isolated as previously described (Jung et al. 2017).

#### Reagents and antibodies

CHIR99021 (S1263), SB15286 (S2729), NaW (10213-10-2), GPI (S0031), NH_4_Cl (A9434), 2-NBDG (72987), and LysoSensor Green DND-189 (L7535) were purchased from Thermo Fisher Scientific. The following primary antibodies were purchased from Cell Signaling Technology: anti-GSK3β (#12456, 1:1000), anti-p-GSK-3β (Ser9) (#5558, 1:1000), anti-Glycogen Synthase (#3886, 1:1000), anti-p-Glycogen Synthase (Ser641) (#47043, 1:1000), anti-iNOS (#13120, 1:1000), anti-arginase1 (#93668, 1:1000), anti-phospho-STAT1-Tyr701 (#9167, 1:1000), anti-STAT1 (#9176, 1:1000), and anti-β-actin (#3700, 1:1000). The following primary antibodies were purchased from BioLegend: anti-human CD68 (333810, 1:100), anti-human CD206 (321104, 1:100), anti-mouse F4/80 (123108, 1:100), and anti-mouse CD206 (141701, 1:100). The following primary antibodies were purchased from Abcam: anti-Ugp2 (ab154817, 1:1000), anti-Pygl (ab190243, 1:1000), anti-TFEB (ab264421, 1:1000), and anti-Col I (ab270993). The primary TMEM175 (19925-1-AP) antibody was purchased from Proteintech. The primary anti-α-SMA (af1032) antibody was purchased from Affinity. The primary anti-Col III (A0817) antibody was purchased from ABclonal.

#### Preparation of mouse macrophages

Bone marrow cells isolated from C57BL/6J mice were cultured for 5 days in complete RPMI-1640 medium containing 20 ng mL^−1^ mouse recombinant macrophage colony-stimulating factor (M-CSF; 315-02, PeproTech). On day 6, bone marrow-derived macrophages (BMDMs) were stimulated with 10 ng mL^−1^ IL-4 (214-14, PeproTech) or 100 ng mL^−1^ LPS plus 20 ng mL^−1^ IFN-γ (315-05, PeproTech) for 24 h to generate anti-inflammatory (M2) or inflammatory (M1) macrophages. Mouse peritoneal macrophages were harvested via peritoneal lavage. Briefly, cold PBS was injected into the peritoneal cavity and extracted after gentle agitation. The peritoneal cell suspension was centrifuged at 1300 rpm, and the cell pellet was mixed with 2 mL of red blood cell lysis buffer for 5 min at room temperature. After washing, the cells were cultured in six-well plates for 3 h. Cells that adhered to the plates were peritoneal macrophages and collected.

#### Real-time PCR

Total RNA extraction was prepared with TRIzol reagent (15596026, Invitrogen), and cDNA was generated with a ReverTra Ace qPCR RT Kit (FSQ-101, Toyobo). Real-time PCR was performed for all genes with primers on a Bio–Rad CFX Connect instrument, and data were captured using Bio–Rad CFX Manager 2.0 software. The expression of mRNA for genes of interest was normalized to the level of *Actb* (Mus) expression. The entire procedure was repeated with at least three biologically independent samples. The primer sequences are shown in Supplementary Table 1.

#### Glycogen level assay

The level of glycogen was measured with a glycogen assay kit (KA0861, Abnova) according to the manufacturer’s instructions.

#### Western blot analysis and ELISAs

Cell lysates and prestained molecular weight markers were separated via SDS‒PAGE, and then, the proteins were transferred to nitrocellulose membranes. The membranes were blocked in Tris-buffered saline with 0.5% Tween 20 (TBST) with 5% bull serum albumin (BSA) and probed with specific antibodies overnight at 4 °C. The membranes were washed three times and incubated with horseradish peroxidase-conjugated secondary antibodies. Immunoreactivity was visualized via enhanced chemiluminescence (ECL) according to a kit manufacturer’s protocol (ECL Kit, 34577, Pierce). Mouse IL-10 (431425, BioLegend), TGFβ (433007, BioLegend), TNF-α (430904, BioLegend) and IL-6 (431307, BioLegend) levels in supernatants were quantified with ELISA kits according to the manufacturer’s protocol.

#### Liquid chromatography–tandem mass spectrometry (LC–MS/MS) analysis

LC–MS/MS analysis was performed on a Q-Exactive mass spectrometer (Thermo Fisher) equipped with a heated electrospray ionization (HESI) probe with the relevant parameters set as follows: heater temperature, 120 °C; sheath gas, 30; auxiliary gas, 10; sweep gas, 3; spray voltage, 2.5 kV in negative mode. A full scan range from 80 to 350 (mz^−1^) was used. The resolution was set to 70,000. The data were captured using Xcalibur™ software, version 3.0 (Thermo Fisher), and quantified by integrating the area underneath the curve of each compound using the Xcalibur Qual browser (Thermo Fisher). Accurate mass ion and subsequent isotopic ion data for each metabolite were extracted using a extracted ion chromatogram (EIC) window of 10 ppm.

#### *Intracellular Ca*^*2*+^*measurement*

Cells were cultured in 24-well plates at a density of 5 × 10^4^ cells/well in RPMI 1640 medium overnight. Briefly, macrophages were stained with 5 μM Fluo-4 AM, and 200 nM ionomycin was used to promote calcium release, as previously described (Wei et al. 2023). After complete cell lysis, the supernatant was centrifuged at 14,000×*g* for 5 min and quantified using a calcium colorimetric kit (S1063S, Beyotime) according to the manufacturer’s protocol.

### RNA sequencing

Two groups of macrophage samples (a Ctrl group and an NaW group, 3 samples each group) were sent to Berry Genomics (Beijing, China) for RNA-seq and bioinformatics analyses.

#### Lysosomal pH value assay

LysoSensor Green is commonly used to qualitatively measure the pH of acidic organelles. The green fluorescence emitted by the probe becomes more intense in increasingly acidic environments and less intense in increasingly alkaline environments. The cells (3 × 10^6^ cells per mL) were loaded with 1 μM LysoSensor Green in prewarmed 1640 medium for 30 min at 37 °C, washed twice with PBS and immediately analyzed via fluorescence microscopy. Quantification of lysosomal pH was performed using the ratiometric lysosomal pH dye LysoSensor Yellow/Blue. A pH calibration curve was generated according to the manufacturer's protocol. Cells were trypsinized and labeled with 10 μM LysoSensor Yellow/Blue for 5 min at 37 °C in 1640 medium and washed with PBS. The labeled cells were treated with 10 μM monensin and 10 μM nigericin for 10 min in 25 mM MES calibration buffer (pH 4.5–7.5) containing 5 mM NaCl, 115 mM KCl and 1.2 mM MgSO_4_. Quantitative comparisons were performed in a 96-well plate, and the fluorescence was measured with a microplate reader (Synergy H1, BioTek) at Ex-360/Em-440 and Ex-360/Em-550.

#### Reactive oxygen species (ROS) level measurements

ROS levels were measured using CellROX Green flow cytometry assay kits (C10444, Invitrogen). Cells were trypsinized and then loaded with 500 nM CellROX Green for 30 min at 37 °C in the dark. The cells were washed with PBS, scraped, maintained in PBS and immediately analyzed using flow cytometry at 488-nm excitation to induce CellROX Green fluorescence.

#### Gene silencing experiments

Short interfering RNAs (siRNAs) targeting mouse *Pygl*, *Tmem175*, and *Tfeb* and negative control siRNAs (NC) were purchased from RiboBio (Guangzhou, China). siRNA (50 nM) was transfected into macrophages using Lipofectamine™ RNAiMAX Transfection Reagent according to the manufacturer’s instructions. The siRNA sequences are shown in Supplementary Table 2.

#### *Intracellular Ca*^*2*+^*measurement*

Cells were cultured in 24-well plates at a density of 5 × 10^4^ cells/well in RPMI 1640 medium overnight. Before Ca^2+^ measurements were taken, cells were washed with PBS 3 times and incubated for 60 min in Hanks’ balanced salt solution containing 5 μM Fluo-4 AM in the dark at room temperature. The cells were then washed with Hanks’ balanced salt solution three times and incubated at room temperature for another 10 min. Then, 200 nM ionomycin was added extracellularly and incubated with the cells for 30 s, and cytosolic calcium release was recorded via low speed by flow cytometry. For an intracellular calcium concentration assay, the cells were washed with PBS and then treated with 100 μL of sample lysate. After cell lysis was completed, the samples were centrifuged for 5 min at 14,000×*g*, and the supernatants were quantified with a calcium colorimetric assay kit (S1063S, Beyotime) according to the manufacturer’s protocol.

#### Wound healing assay

In brief, the wound healing technique involved creating a thin linear scratch “wound” (creating a gap) in a confluent monolayer of cells. Periodically, images of the cells were taken, and the reduction in the gap width was measured as described previously (Grada et al. 2017).

#### Statistical analysis

All experiments were performed at least three times. The results are expressed as the mean ± SEM and were analyzed by two-tailed unpaired Student’s *t* test or one-way ANOVA. In all tests, *p* values of less than 0.05 were considered statistically significant. The analysis was conducted using GraphPad Prism 8.0 software.

### Data availability

The authors declare that the data supporting the findings of this study are available within the article and its Supplementary Information files or from a corresponding author on reasonable request. The raw data used to generate the figures and supplementary figures are presented in a Source Data file.

## Results

### M2 macrophages are the main components of macrophages in CF

**Fig. 1:**
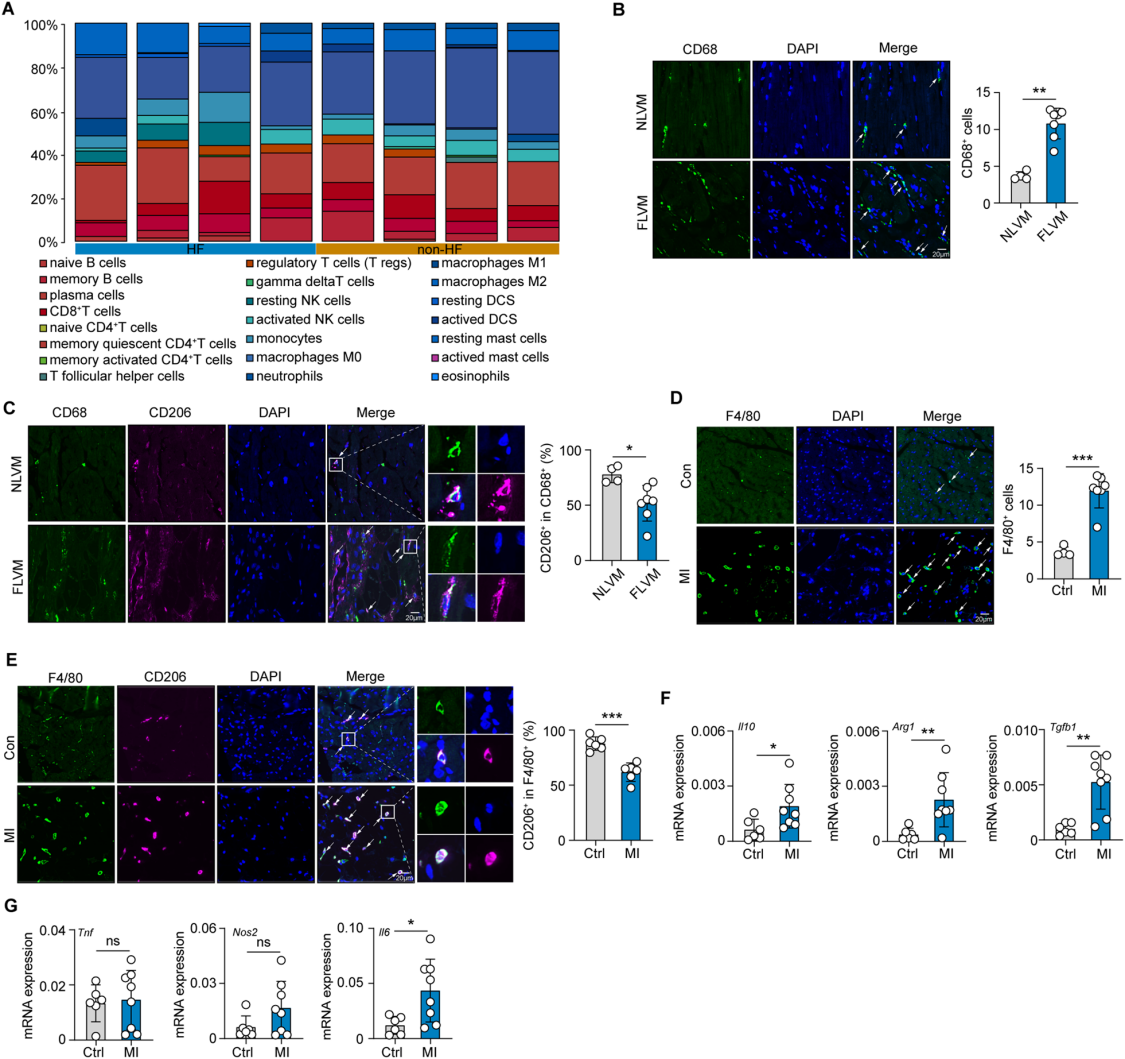
M2 macrophages are the main macrophage type in cardiac fibrotic tissues. **A** Boxplots show the proportions of various types of immune cells infiltrated that infiltrated tissues from people with and without heart failure (HF). **B**, **C** Representative immunofluorescence images and quantitative analysis of CD68^+^ (**B**), CD68^+^ CD206^+^ and CD68^+^ CD206^−^ (**C**) macrophages in human fibrotic left ventricular myocardium (FLVM) (n = 7) and normal left ventricular myocardium (NLVM) (n = 4). Green, CD68; pink, CD206; blue, DAPI. Scale bar, 20 μm. Three microscope images/sample. **D**, **E** Representative immunofluorescence images and quantitative analysis of F4/80^+^ (**D**), F4/80^+^ CD206^+^ and F4/80^+^ CD206^−^ (**E**) macrophages in the fibrotic area of mice after MI (n = 6) and control mice (n = 6). Green, F4/80; pink, CD206; blue, DAPI. Scale bar, 20 μm. Three microscope images/sample. **F**, **G** On the 38th day after MI induction, F4/80^+^ macrophages were isolated from the fibrotic tissues of MI-induced (n = 8) and control (n = 6) mice, and the expression of *Il10*, *Arg1*, *Tgfb1*, *Tnf*, *No2*, and *Il6* in macrophages was measured by real-time PCR. Unless otherwise specified, n = 3 biologically independent experiments. The data are presented as the mean ± SEM. P values were calculated by one-way ANOVA. *P < 0.05; **P < 0.01; ***P < 0.001

Previous reports have shown that macrophages play important roles in CF (Revelo et al. 2021; Hulsmans et al. 2018; Kong et al. 2014). In this study, we applied a multistep integrated bioinformatics analysis of HF and non-HF myocardial tissue to identify infiltrating immune cell populations and important signaling pathways that may play a profibrotic role. First, the CIBERSORT algorithm was used to estimate the relative proportions of 22 immune cell subtypes based on bulk RNA-Seq data (from the GSE76701 dataset), which included those from 8 samples (PMID: 26756417). These cells included macrophage subsets (M0, M1 and M2 macrophages), T cells (CD8^+^, naive CD4^+^ T cells, memory quiescent CD4^+^ T cells, memory activated CD4^+^ T cells, T follicular helper cells, regulatory T cells and gamma delta T cells), natural killer (NK) cells (resting and activated NK cells), mast cells (resting and activated mast cells), B cells (naive and memory B cells), dendritic cells (DCs; resting and activated DCs), monocytes, plasma cells, eosinophils, and neutrophils (Supplementary Fig. 1A). In addition, the proportion of M2 macrophages was the largest among the infiltrated immune cells (Fig. 1A). We also defined 11 clusters of macrophages according to their known markers (Supplementary Fig. 1B) (GSE161470) (PMID: 34088950). At the same time, functional enrichment analyses were performed in macrophage differentially expressed genes (DEGs) between humans with and healthy humans without HF (Supplementary Fig. 1C). As shown in supplementary Fig. C, TGF-β signaling, a major CF-promoting pathway (Khalil et al. 2017), was found to be enriched via a Kyoto Encyclopedia of Genes and Genomes (KEGG) analysis. M2 macrophages are important sources of TGFβ. The aforementioned analyses and results suggest that M2 macrophages may be an important factor in promoting the development of CF. Therefore, we analyzed macrophage phenotypes in seven FLVM and four NLVM tissue samples. We found that compared with that in NLVM samples, the number of CD68^+^ macrophages in the FLVM samples was significantly increased, and this cell subset comprised mainly CD68^+^CD206^+^ M2 macrophages (Fig. 1B, C), and the number of CD206^+^ M2 macrophages in the FLVM samples was higher than that in NLVM, although the proportion of M2 macrophages was slightly higher in the tissues from people without HF (Supplementary Fig. A). Subsequently, we induced CF by subjecting mice to MI. Compared with that in the normal control mice, the infiltration of F4/80^+^ macrophages was significantly increased in the myocardial fibrotic tissues of the MI model mice (Fig. 1D), and this cell subset comprised mainly F4/80^+^CD206^+^ M2 macrophages (Fig. 1E). In addition, significantly more F4/80^+^CD206^+^ M2 macrophages were found in the fibrotic tissues of the MI model mice than in normal control group mice (Fig. 1E). These findings were similar to those observed in human cardiac fibrotic tissues. To further understand the phenotype of the macrophages in cardiac fibrotic tissues, we isolated F4/80^+^ macrophages and found that compared with those in the normal control group, the levels of interleukin 10 (*Il10*, a profibrotic factor) (Hulsmans et al. 2018), transforming growth factor beta 1 (*Tgfb1*, a profibrotic factor), and arginase 1 (*Arg1*, an M2 macrophage marker) was increased in the macrophages in the myocardial fibrotic tissues (Fig. 1F); the levels of the proinflammatory factors tumor necrosis factor (*Tnf*) and nitric oxide synthase 2 (*Nos2,* a marker of M1 macrophages) were not different (Fig. 1G); and the interleukin 6 (*Il6*) levels were significantly increased and may be related to CF promotion (Kumar et al. 2019; Mia et al. 2020) (Fig. 1G). Together, these findings suggest that M2 macrophages are the main components of macrophages in the CF context and may play important roles in the development of CF.

### Reprogramming macrophage metabolism into that of M1 macrophages

**Fig. 2:**
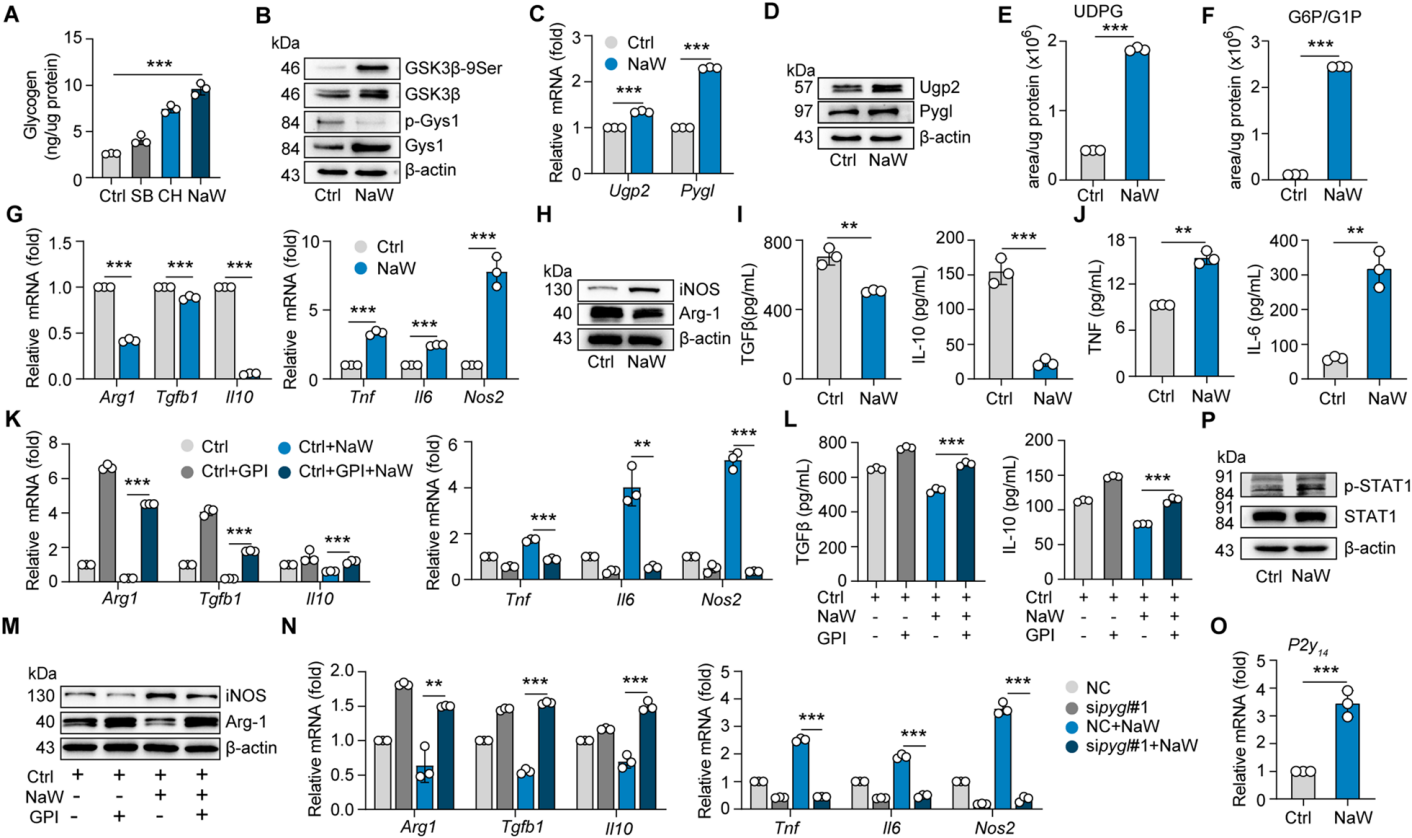
Reprogramming macrophage metabolism into that of M1 macrophages. Mouse bone marrow-derived macrophages (BMDMs) were harvested and treated with IL-4 (10 ng mL^−1^) for 12 h to induce M2 macrophage switching. **A** The levels of intracellular glycogen in M2 macrophages treated with CHIR99021:CH (3 μM), SB415286:SB (3 μM) or NaW (2 mM) were measured by colorimetric assay. **B** The protein levels of phosphorylated GSK3β-9Ser, GSK3β, p-Gys1 and Gys1 were measured by Western blotting. **C**, **D** The expression of *Ugp2* and *Pygl* in M2 macrophages with or without NaW treatment was analyzed by real-time PCR (**C**) and (**D**) western blotting. **E**, **F** Liquid chromatography–tandem mass spectroscopy** (**LC‒MS/MS) was performed for mearing UDPG (**E**) and G6P/G1P (**F**) levels in M2 macrophages treated with or without NaW. **G**–**J** The expression of *Arg1*, *Tgfβ1*, *Il10*, *Tnf*, *Il6* and *Nos2* in M2 macrophages with or without NaW treatment was analyzed by real-time PCR (**G**). iNOS, Arg-1, TGFβ, IL-10, TNF and IL-6 levels were measured by western blotting (**H**) and ELISAs (**I**, **J**). **K**‒**M** M2 macrophages were pretreated with a glycogen phosphorylase inhibitor (GPI) for 6 h. The expression levels of *Arg1*, *Tgfβ1*, *Il10*, *Tnf*, *Il6* and *Nos2* in M2 macrophages treated with or without NaW were measured by real-time PCR (**K**), IL-10, TGFβ, IL-6 and TNF expression was measured by ELISAs (**L**), and iNOS protein expression was measured by western blotting (**M**). **N** The expression of *Arg1*, *Tgfβ1*, *Il10*, *Tnf*, *Nos2* and *Il6* in M2 BMDMs pretransfected with siRNA (Pygl) and treated with or without NaW before IL-4 stimulation as measured by real-time PCR. **O**, **P** The expression of *P2Y*_*14*_ in M2 macrophages treated with or without NaW was measured by real-time PCR (**O**), and the expression of STAT1 and p-STAT1 was measured by western blotting (**P**). Unless otherwise specified, n = 3 biologically independent experiments. The data are presented as the mean ± SEM. P values were calculated by one-way ANOVA. *P < 0.05; **P < 0.01; ***P < 0.001

In the early inflammatory phase after tissue injury and MI, activation of M1 macrophages leads to pathogenic microorganism removal and extracellular matrix degradation mediated through extracellular matrix metalloproteinases (MMPs), which release proinflammatory cytokines to promote inflammation (Lawrence and Natoli 2011; Ma et al. 2018; Horst et al. 2015). In contrast, anti-inflammatory M2 macrophages are characterized by the overexpression of TGFβ, IL10, Arg1 and other signature proteins, which are associated with matrix deposition, promoting CF (Ma et al. 2018; Horst et al. 2015; Peet et al. 2020; Yang et al. 2018). To explore the possibility that M2 macrophages polarize into M1 macrophages and thus attenuate CF, we based a previous study on the key role of glycogen metabolism in memory T-cell formation, the maintenance of CD8^+^ T cells (Zhang et al. 2020), and M1 polarization of macrophages (Ma et al. 2020). We targeted glycogen synthase kinase 3β (GSK3β) to enhance glycogen metabolism of macrophages to be similar to that in M1 macrophages. GSK3β is a key upstream molecule that regulates the glycogen metabolism cycle. It promotes the phosphorylation of glycogen synthase 1 (Gys1) through its kinase activity, and the inactivation of phosphorylated Gys1 leads to a reduction in glycogen synthesis. Therefore, we identified several small-molecule compounds and inhibitors that target GSK3β: CHIR99021 (Buikema 2020), SB415286 (Torre et al. 2012) and NaW (Gómez-Ramos et al. 2006). By comparing the effects of these three GSK3β inhibitors on glycogen synthesis in M2 macrophages, we found that CHIR99021, SB415286 and NaW increased the glycogen level of M2 macrophages, and NaW exerted the greatest effect (Fig. 2A). NaW effectively promoted the phosphorylation of GSK3β-Ser9 and further inhibited the phosphorylation of Gys1 in M2 macrophages (Fig. 2B). Consistent with these results, we found that the expression of other enzymes involved in glycogen biosynthesis and breakdown, including UDP-glucose pyrophosphorylase 2 (Ugp2, a catalytic enzyme involved in the conversion of G1P to UDPG) and glycogen phosphorylase Pygl (a catalytic enzyme required for glycogenolysis), was upregulated in M2 macrophages after NaW treatment (Fig. 2C, D). Moreover, the levels of UDPG and G6P/G1P were significantly increased after NaW treatment compared to those in M2 macrophages, as determined by LC–MS/MS analysis (Fig. 2E, F). In addition, NaW effectively reduced the levels of the profibrotic factors TGFβ and IL-10 and the marker Arg1 in M2 macrophages while increasing the levels of the inflammatory factors TNFα, IL-6 and iNOS (Fig. 2G–J). Meanwhile, in comparison to the pre-withdrawal period, there were no statistically significant alterations observed in the macrophage phenotype at 24 and 48 h following NaW withdrawal (Supplementary Fig. 2A–D). This implies that, in the absence of other inducing factors, short-term changes in macrophage phenotype may not be substantial. When glycogen metabolism was blocked by a glycogen phosphorylase inhibitor (GPI) and an siRNA directed against *Pygl*, the effect of NaW on macrophage phenotypic conversion was inhibited (Fig. 2K–N). These results suggest that NaW promotes the conversion of M2 macrophages to M1 macrophages by enhancing the glycogen synthesis and decomposition pathways. Our previous report showed that glycogen-derived UDPGs activate the P2Y_14_ receptor, whose signaling modulates inflammatory phenotype acquisition by macrophages in an autocrine manner (Ma et al. 2020). Indeed, the levels of P2Y_14_ and phosphorylated STAT1, a key transcription factor that mediates the switch to an macrophage inflammatory phenotype and is activated after phosphorylation, were increased in M2 macrophages treated with NaW (Fig. 2O, P). In addition, the levels of pentose phosphate pathway (PPP)-related enzymes G6P dehydrogenase (G6pdx) and 6-phosphogluconate dehydrogenase (6Pgd) were also increased in M2 macrophages treated with NaW (Supplementary Fig. 2E, F). However, intracellular ROS level were decreased (Supplementary Fig. 2G), which indicated that NaW treatment protected M2 macrophages from damage. Together, the data suggest that NaW enhances glycogen metabolism in M2 macrophages and promotes their conversion to be macrophages with an inflammatory phenotype.

### NaW activates inflammation gene expression and glucose transport in M2 macrophages

**Fig. 3:**
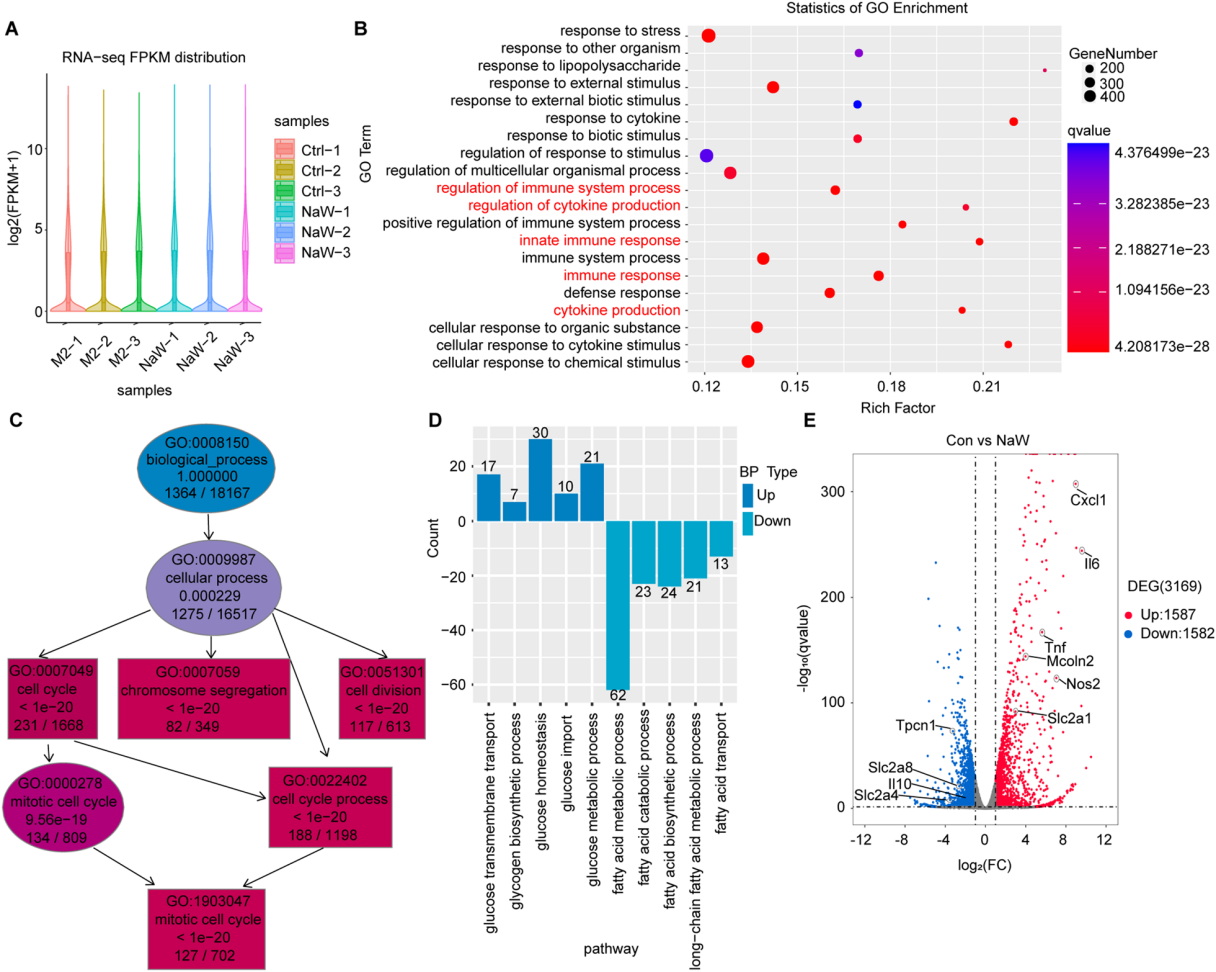
NaW activates inflammatory and glucose transport genes in M2 macrophages. Six samples of IL-4-conditioned bone marrow-derived macrophages (BMDMs) with or without NaW (2 mM) treatment (Ctrl-1, Ctrl-2, Ctrl-3, NaW-1, NaW-2, or NaW-3) were collected for RNA-seq analysis. **A** Violin plot shows the abundance of genes expressed in the 6 samples. **B**, **C** GO results are represented using bubble plots (**B**) and directed acyclic graphs (**C**). **D** KEGG results are presented in a bar plot. **E** DEGs in a volcano plot

To further explore the effect of NaW on the mechanism underlying its effect on glucose metabolism in M2 macrophages, two groups of macrophage samples (a Ctrl group and NaW group, with 3 samples per group) were selected for RNA-seq analysis. As shown in Fig. 3A, based on the fragments per kilobase of exon model per million mapped fragments (FPKM) values of all genes in each sample, violin plots showed the abundance distribution characteristics of RNA-seq gene expression for each sample, and slightly higher gene expression levels were observed for the NaW group compared to the Ctrl group (Fig. 3A). Gene Ontology (GO) and KEGG functional enrichment analyses of DEGs showed that the biological process (BP) category terms were related mainly to immune response and regulation. These terms included regulation of the immune system process, regulation of cytokine production, innate immune response, immune response, and cytokine production (Fig. 3B and Supplementary Fig. 3A). However, in the BP category, cell division and proliferation gene expression was inhibited; the relevant BP terms included cell cycle, chromosome segregation, and cell division (Fig. 3C and Supplementary Fig. 3B). These findings confirmed that NaW activates the inflammatory response of macrophages but inhibits macrophage proliferation and division. KEGG enrichment analysis showed that pathways related to glucose metabolism were activated at a significantly higher level, while the pathways related to fatty acid metabolism were significantly inhibited (Fig. 3D). Batch RNA-seq with 6 macrophage samples (3 Ctrl vs. 3 NaW) led to the identification of 3169 DEGs, of which 1587 genes were upregulated and 1582 genes were downregulated. Among these DEGs, the expression levels of inflammatory factors such as *IL6*, *Tnf*, and *Nos2* and the transcript level of solute carrier family 2 member 1 (*Slc2a1*) were increased, while the transcript levels of *IL10*, *Slc2a4*, and *Slc2a8* were decreased. Mucolipin TRP cation channel 2 (Mcoln2) expression was upregulated (Fig. 3E and Supplementary Fig. 3C). These findings suggest that NaW may promote glucose transport in macrophages and provide raw materials needed for glycogen synthesis; the related mechanisms need to be further explored.

### ***NaW increases lysosomal pH-mediated Ca***^***2***+^***release and thus enhances glucose uptake***

**Fig. 4:**
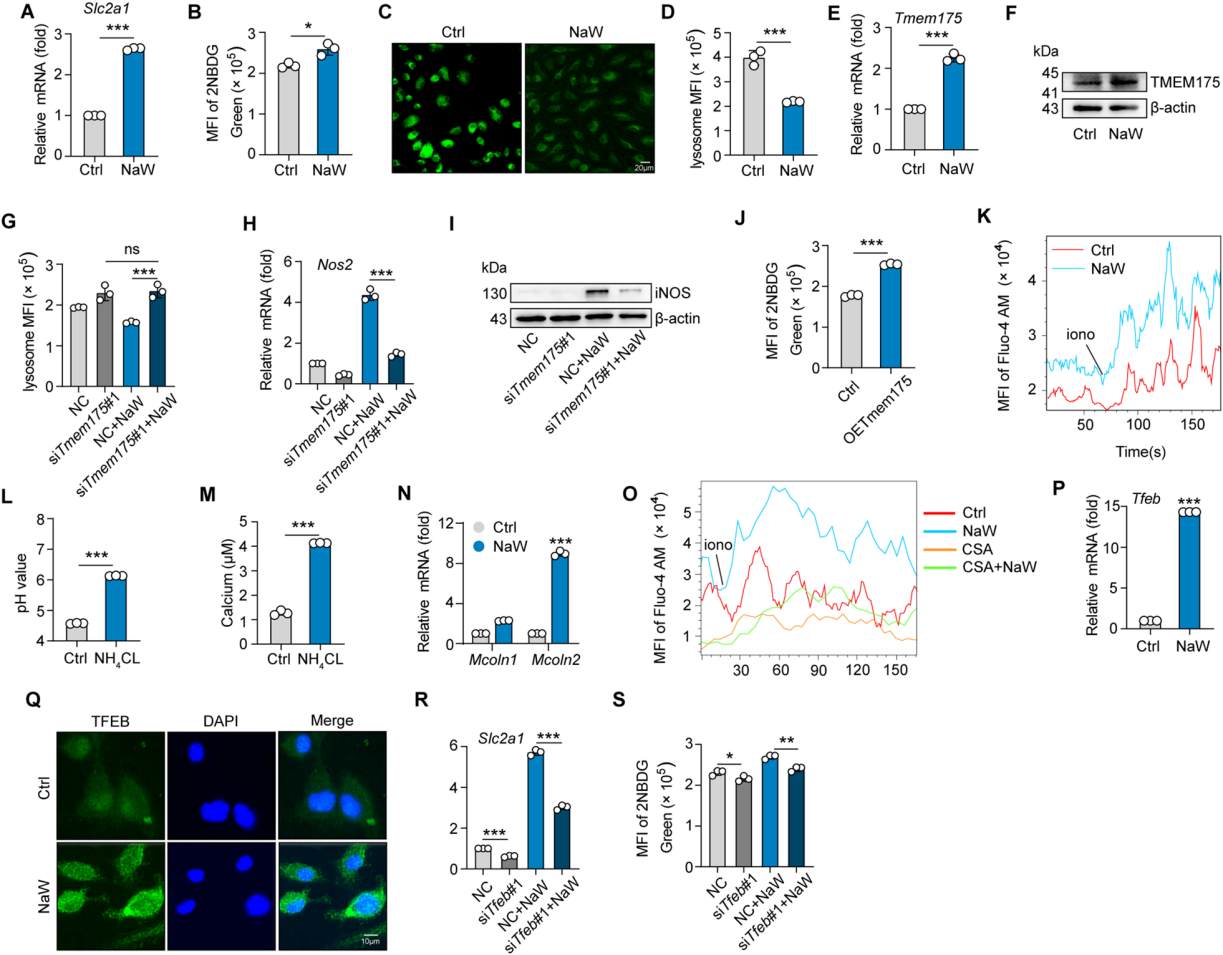
NaW increases lysosomal pH-mediated Ca^2+^ release and thus enhances glucose uptake. **A**–**G** IL-4-conditioned bone marrow-derived macrophages (BMDMs) were treated with or without NaW (2 mM) for 12 h. **(A)** Scl2a1 expression was measured by real-time PCR. **B** The mean fluorescence intensity (MFI) level of 2NBDG was measured by flow cytometry. **C** LysoSensor Green-labeled acidic lysosomes were observed under a fluorescence microscope. Scale bar, 20 μm. **D** The MFI of the LysoSensor probe was measured by flow cytometry. **E**, **F** *TMEM175* expression was measured by real-time PCR (**E**) and western blotting (**F**). **G**–**I** IL-4-conditioned BMDMs were transfected with *TMEM175* siRNAs for 12 h, the MFI of the LysoSensor probe was detected by flow cytometry (**G**), and *Nos2* expression was determined by real-time PCR (**H**) and western blotting (**I**). **J** After *Tmem175* overexpression in macrophages, the MFI emitted by 2NBDG was measured by flow cytometry. **K** IL-4-conditioned BMDMs were treated with or without NaW (2 mM) for 12 h, and cytosolic calcium release was measured by flow cytometry. **L**, **M** IL-4-conditioned BMDMs were treated with NH_4_Cl for 5 min, the pH value of lysosomes was measured with a microplate reader (**L**), and the cytosolic calcium release rate was measured by flow cytometry (**M**). **N** The expression of Mcoln1 and Mcoln2 was analyzed by real-time PCR. **O** The same as presented in (**A**), except cells were pretreated with 10 nM CsA for 1 h. **P**, **Q** The same as presented in (**A**), except the expression and location of TFEB were analyzed by real-time PCR (**P**) and immunofluorescence (**Q**) (scale bar, 20 μm). **R**, **S** IL-4-conditioned BMDMs were transfected with *Tfeb* siRNA, *Scl2a1* expression was measured by real-time PCR, and the MFI of 2NBDG was measured by flow cytometry. Unless otherwise specified, n = 3 biologically independent experiments. The data are presented as the mean ± SEM. P values were calculated using one-way ANOVA. *P < 0.05; **P < 0.01; ***P < 0.001

Next, the effects of NaW on the glucose uptake capacity of macrophages and the underlying mechanisms were investigated. We found that *Slc2a1* expression was upregulated in M2 macrophages after NaW treatment (Fig. 4A), and the expression of *Slc2a2*, *Slc2a4* and *Slc2a8* was decreased (Supplementary Fig. 4A). At the same time, NaW promoted the uptake of the glucose fluorescent analog 2NBDG in macrophages, indicating that the glucose uptake ability of these macrophages was enhanced (Fig. 4B). As professional phagocytes, macrophages show a clear ability to absorb extracellular substances and efficiently degrade them in lysosomes. This degradation process strictly depends on the pH value of acidic lysosomes (Casey et al. 2010; Appelqvist et al. 2013). Our previous study showed that changing the lysosomal pH may be a potential strategy to drive macrophage phenotype switching by changing the glucose metabolism program (Chen et al. 2018). As an alkalinizing drug, NaW increases lysosomal pH (Fig. 4C, D). Gene and protein expression of TMEM175, a lysosomal ion channel that mediates H^+^ efflux, was increased (Fig. 4E, F), while the expression of V-ATPase (an ion pump mediating H^+^ transport into lysosomes) did not change (Supplementary Fig. 4B). After we knocked down *Tmem175* (Supplementary Fig. 4C), the lysosomal pH value and iNOS expression level returned to baseline after NaW treatment (Fig. 4G–I). In addition, *Tmem175* overexpression (Supplementary Fig. 4D) in macrophages promoted the uptake of 2NBDG by macrophages, indicating that their glucose-uptake ability was enhanced by 2NBDG (Fig. 4J). These findings suggest that NaW increases lysosomal pH and the glucose transport rate by changing TMEM175 expression. Because lysosomes are acidic Ca^2+^ reservoirs (Christensen et al. 2002), an increase in lysosomal pH increases triggers the release of lysosomal Ca^2+^ into the cytoplasm and activate macrophages (Chen et al. 2018; Li et al. 2018). NaW treatment increased the M2 macrophage Ca^2+^ level by increasing the lysosomal pH (Fig. 4K). Ammonium chloride (NH_4_Cl) rapidly increases lysosomal pH (Girón et al. 2008). Following the NH_4_Cl treatment for 1–2 min, increases in lysosomal pH and cytosolic Ca^2+^ were observed, which was similar to the increase induced by NaW treatment. (Fig. 4L, M). Furthermore, we found that the expression of the major lysosomal channel Mcoln2, a Ca^2+^ channel that mediates lysosomal calcium release, was upregulated after NaW treatment. However, the *Tpcn1* and *Tpcn2* expression levels were basically unchanged (Fig. 4N and (Supplementary Fig. 4E). In addition, the Ca^2+^ signaling inhibitor cyclosporin A (CsA) decreased the aforementioned Ca^2+^ release from lysosomes (Fig. 4O). Collectively, these results suggest that increasing lysosomal pH promotes the lysosomal Ca^2+^ release rate. M1 polarization of macrophages not only results in phenotype alterations (changes in surface marker, cytokine, and enzyme levels) but also leads to reprogrammed metabolism (Nomura et al. 2016; Pearce and Pearce 2013; Huang et al. 2014). Reportedly, activation of the intracellular Ca^2+^-TFEB pathway regulates the intracellular glucose supply and promotes glucose metabolism in macrophages (Chen et al. 2018). We found that both TFEB expression and nuclear localization were upregulated in response to NaW treatment (Fig. 4P, Q). Knocking down *Tfeb* by siRNAs (Supplementary Fig. 4F) led to the downregulation of Slc2a1 expression and reduced 2NBDG uptake (Fig. 4R, S). Collectively, these results suggest that increasing lysosomal pH promotes lysosomal Ca^2+^ release, which triggers alterations to metabolic patterns in macrophages.

### NaW inhibits CF and promotes macrophage phenotype conversion

**Fig. 5:**
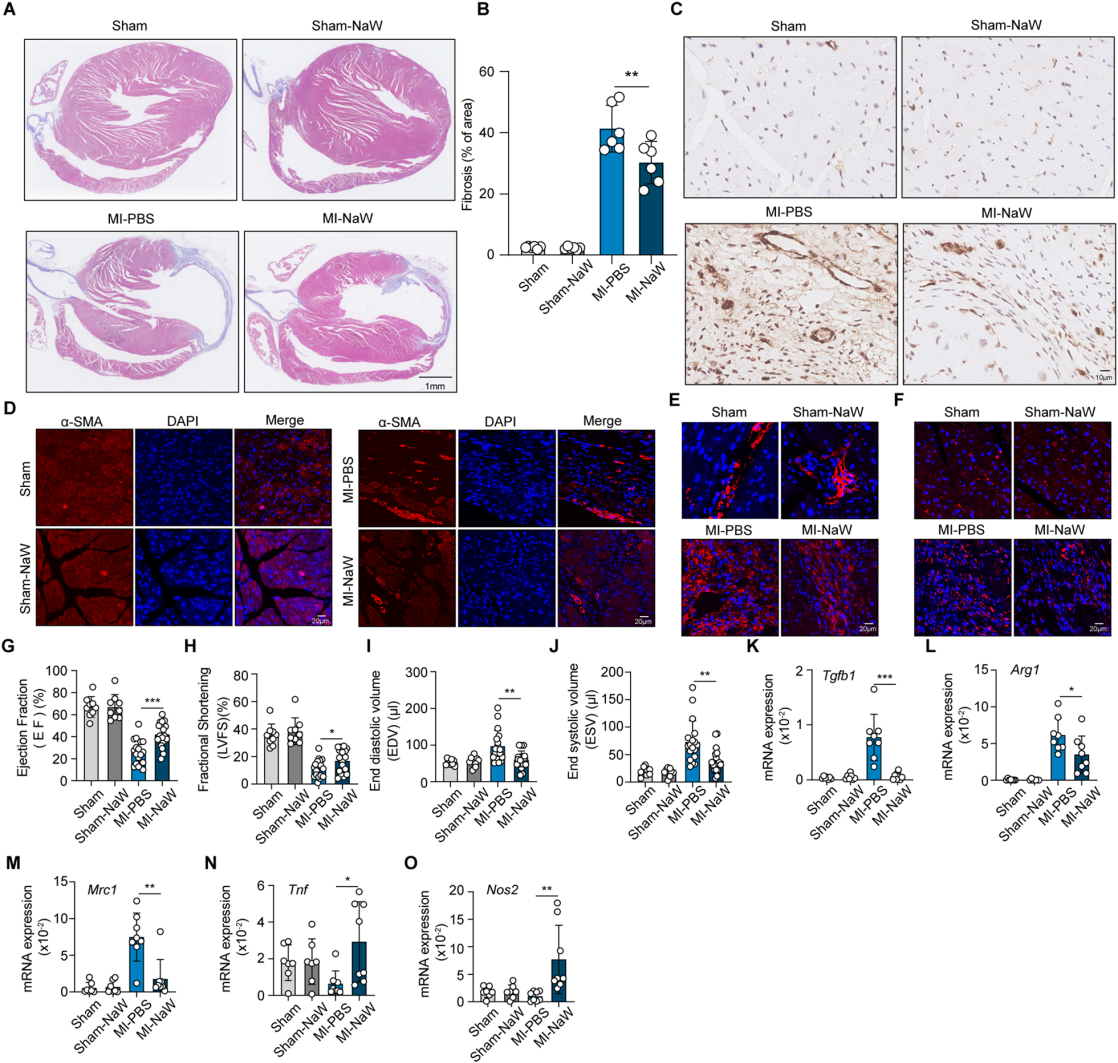
NaW inhibits CF and promotes macrophage phenotype conversion. **A**, **B** Thirty-eight days after the sham/MI operation, the hearts of sham, sham-NaW, MI-PBS and MI-NaW mice were observed by Masson’s trichrome staining and microscopy (scale bar, 1 mm) (**A**), and the fibrotic region areas were quantified (**B**), n = 6. **C**, **D** Representative immunostaining (scale bar, 10 μm) (**C**) and immunofluorescence (scale bar, 20 μm) (**D**) for α-SMA in fibrotic regions of the heart 38 days after the sham/MI operation. **C** Blue: hematoxylin; brown: a-SMA. **D** Red: a-SMA; blue: DAPI. **E**, **F** Representative images showing collagen I (**E**) and III (**F**) immunofluorescence staining. Scale bar, 20 μm. **G**–**J** Ejection fraction (EF) (**G**), left ventricular fractional shortening (LVFS) (**H**) end-diastolic volume (EDV) (**I**) and end systolic volume (ESV) (**J**) as quantified via echocardiography 38 days after the sham/MI operation; n = 9, 9, 19, and19, respectively. **K**–**O** On the 38th day after MI surgery, F4/80^+^ macrophages were isolated from the fibrotic area of MI-PBS, MI-NaW, sham, and sham-NaW mice, and the expression of *Tgfb1*, *Arg1*, *Mrc1*, *Tnf*, and *Nos2* in macrophages was measured by real-time PCR; n = 7, 7, 8, and 8, respectively. **P**–**R** The expression of *Gys1*, *Ugp2*, *Slc2a1*, and *Pygl* in the M2 macrophages isolated from the hearts of two groups of mice was measured by real-time PCR. n = 8, 8, 8, and 8, respectively. Unless otherwise specified, n = 3 biologically independent experiments. The data are presented as the mean ± SEM. P values were calculated by one-way ANOVA. *P < 0.05; **P < 0.01; ***P < 0.001

NaW, a xanthine oxidase inhibitor, has shown good diabetes and obesity treatment effects in different type 1 and type 2 diabetic animal models (Amigó-Correig et al. 2011; Nocito et al. 2012; Muñoz et al. 2001), and relevant clinical trials have been conducted (Hanzu et al. 2010). Some studies have shown that xanthine oxidase inhibitors effectively attenuate CF (Engberding et al. 2004; Sagor et al. 2015). Therefore, we investigated the effect of NaW on CF. Following previous reports, we performed in vivo and in vitro experiments with 100 mg kg^−1^ and 2 mM NaW (Gómez-Ramos et al. 2006; Girón et al. 2008; Aydemir et al. 2012), respectively. After approximately 10 days of cardiac repair in mice after MI induction, NaW was administered by gavage for 28 days, and we found that CF was inhibited in the treatment group compared with the effects in the MI group (Fig. 5A, B), and the number of α-SMA^+^ myofibroblasts was decreased (Fig. 5C, D). Moreover, immunofluorescence staining of collagen I and III showed that interstitial fibrosis in the mice of the MI-NaW group had been alleviated compared with that in the MI-PBS mice group (Fig. 5E, F). Moreover, after treatment MI model mice with NaW, the ejection fraction (EF), left ventricular fractional shortening (LVFS), end-diastolic volume (EDV) and end systolic volume (ESV) were improved (Fig. 5G–J). We also found that the expression of proinflammatory factors increased and anti-inflammatory factors decreased in cardiac macrophages (Fig. 5K–O), suggesting that the macrophage phenotype had polarized toward the M1 phenotype. The density of macrophages in the fibrotic areas, however, did not exhibit any significant alteration (Supplementary Fig. 5A). To further validate the impact of NaW on the glycogen metabolic synthesis pathway in cardiac macrophages of MI mice, we isolated M2 macrophages from the hearts of both groups (3 MI-PBS and 3 MI-NaW) and conducted RNA-seq. KEGG functional enrichment analysis of GO and DEGs showed that they were mainly related to the activation of glucose metabolism and inflammatory signaling pathways (Supplementary Fig. 5B, C). A batch RNA-seq was performed on 6 macrophage samples, and 1132 DEGs were identified, of which 624 genes were up-regulated and 508 genes were down-regulated. In these DEGs, the expression levels of inflammatory factors such as *Il6* and *Ccl2* and the up-regulated transcription levels of solute vector family member 1 (*Slc2a1*) and Mucolipin TRP cation channel 2 (*Mcol2*) were increased, while the transcription levels of *Tgfbr1* and *Tpcn1* were decreased (Supplementary Fig. 5D). The F4/80^+^ CD206^+^ M2 macrophages were simultaneously isolated for qPCR analysis. In comparison to MI-PBS, the expression levels of *Gys1*, *Ugp2*, and *Slc2a1* genes were found to be up-regulated in the MI-NaW group (Fig. 5P–R). Additionally, there was a slight up-regulation in the expression level of *Pygl*; however, it did not reach statistical significance (Supplementary Fig. 5E). The overall effect of NaW is a potent inhibition of CF, promotion of macrophage glycogen metabolism in myocardial fibrosis, and induction of their polarization towards M1 macrophages, which aligns closely with our in vitro research findings. Together, these results suggest that NaW inhibits CF and reprograms macrophages to differentiate, enabling them to switch from acquiring a profibrotic phenotype to acquiring an inhibitory fibrotic phenotype.

### Inhibition of fibroblast activation by NaW depends on macrophage phenotype conversion

**Fig. 6:**
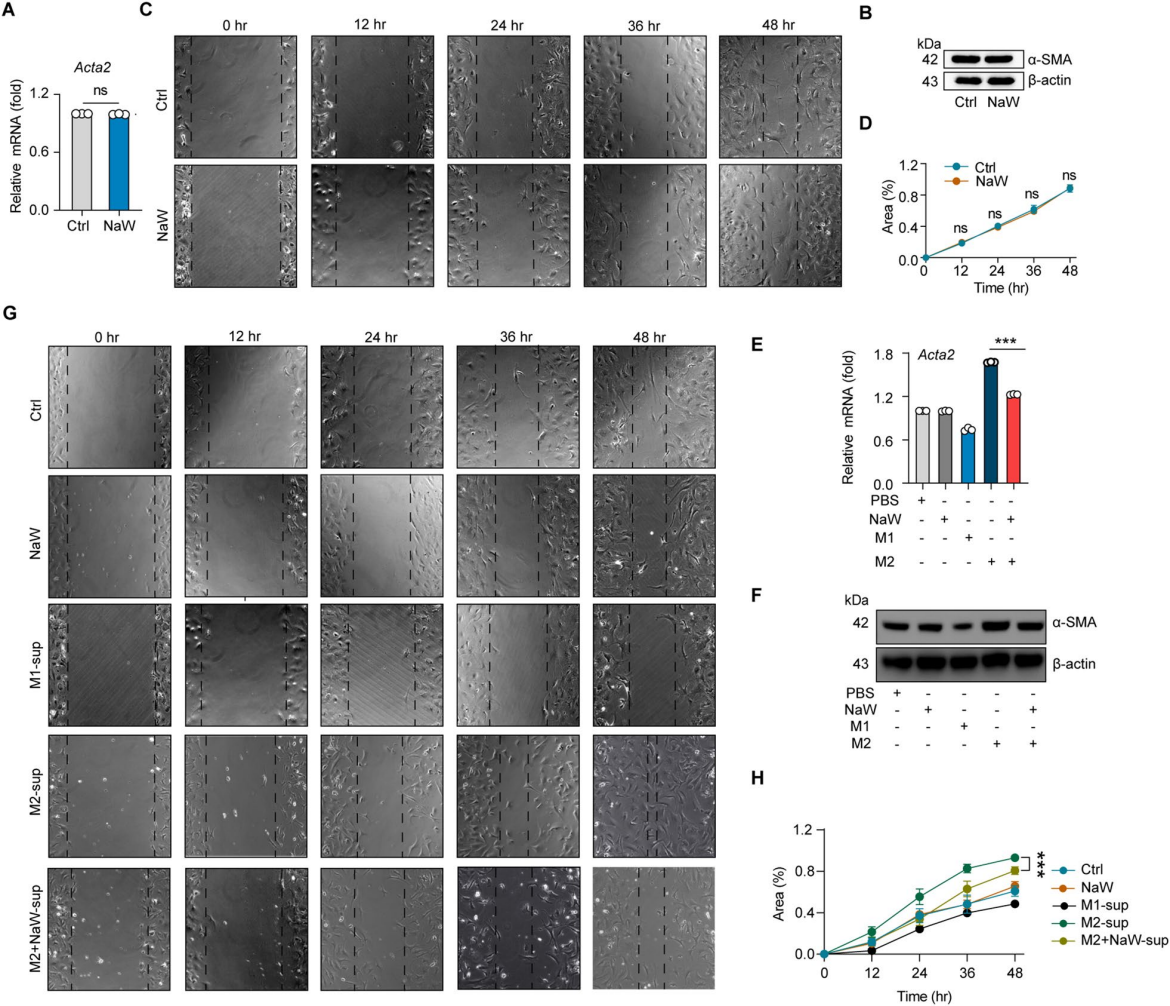
Inhibition of fibroblast activation by NaW is dependent on macrophage phenotype conversion. **A**, **B** Fibroblasts were activated with TGFβ for 48 h and treated with PBS and NaW (2 mM). The α-SMA expression levels in fibroblasts was measured by real-time PCR (**A**) and western blotting (**B**). **C**, **D** Fibroblasts were activated with TGFβ for 48 h and treated with PBS (Ctrl) and NaW (2 mM). The proliferation of fibroblasts and the degree to which they migrated were observed (**C**) and quantified (**D**) via wound-healing assay, n = 6 and 7, respectively. **E**, **F** Fibroblasts were activated with TGFβ for 48 h and treated with PBS (Ctrl), NaW, M1-sup, M2-sup or M2 + NaW-sup. The α-SMA expression levels in fibroblasts was measured by real-time PCR (**E**) and western blotting (**F**). **G**, **H** Fibroblasts were activated with TGFβ for 48 h and treated with PBS (Ctrl), NaW, M1-sup, M2-sup or M2 + NaW-sup. The proliferation of fibroblasts and the degree to which they migrated were observed (**G**) and quantified (**H**) by wound healing assay; n = 7, 7, 5, and 5, respectively. Unless otherwise specified, n = 3 biologically independent experiments. The data are presented as the mean ± SEM. P values were calculated by one-way ANOVA. *P < 0.05; **P < 0.01; ***P < 0.001

Next, we investigated the pathway underlying the inhibitory of NaW on CF. Activated fibroblasts transition into myofibroblasts and secrete excessive levels of collagen into the intercellular stroma, which are important processes leading to CF (Frangogiannis 2021; Eguchi 2021). Therefore, we treated fibroblasts after TGFβ activation with PBS and NaW in vitro, and we found that the expression of α-SMA, a marker of fibroblast activation, had not been affected (Fig. 6A, B). In addition, no difference in the migration or proliferation capacity of the activated fibroblasts between the two groups was found, as determined by wound healing assay (Fig. 6C, D). These findings suggested that NaW did not directly inhibit the fibroblast activation. Because of the important role played by macrophages in CF, we next investigated the relationship between the conversion of M2 macrophages into M1 macrophages and fibroblast activation after NaW treatment. Specifically, M2 macrophages were treated with NaW, and the supernatant from these cultured cells was used to treat fibroblasts while TGFβ was activated (M2 + NaW-sup group). Compared with that in the M2 macrophage supernatant treatment group (M2-sup group), the expression of α-SMA in the M2 + NaW-sup group was decreased (Fig. 6E, F), and the migration and proliferation capacities of the M2 macrophages were significantly decreased (Fig. 6G, H). This suggested that NaW inhibits fibroblast activation by inducing M1 macrophage polarization. In conclusion, inhibition of CF by NaW depends on macrophage phenotype change.

## Discussion

CF is caused by excessive activation of cardiac fibroblasts, which transition into myofibroblasts and secrete a large amount of collagen and deposit in the heart interstitium (Porter and Turner 2009). CF involves a common pathological change in the end stage of a variety of cardiovascular diseases, and most recent studies have targeted CF treatments directly to myofibroblasts (Travers et al. 2022; Park et al. 2019). It was previously thought that myofibroblasts differentiated from fibroblasts, but now more and more studies have shown that the relationship between myofibroblasts and fibroblasts is much more complex. In addition, scRNAseq and lineage-tracing techniques have demonstrated the heterogeneity of fibroblasts. Therefore, effective therapies for CF are eagerly awaited, and analysis of cell signaling pathways in various fibroblast states will help identify therapeutic targets suitable for drug development (Kurose 2021). Although activated myofibroblasts are the primary effector cells in CF tissues, monocytes/macrophages, lymphocytes, mast cells, vascular cells, and cardiomyocytes may also be involved in fibrosis by secreting key fibrogenic mediators (Frangogiannis 2021). For instance, in the process of MI, the regenerative capacity of cardiomyocytes is severely limited and falls short of meeting the extent of damage caused by MI. At the same time, the immune response activates the inflammatory response and fibroblasts, promoting scar repair and cardiac hypertrophy to compensate for the repair of heart function and the maintenance of heart integrity. However, an uncontrolled immune response will cause excessive extracellular matrix deposition, promoting the occurrence of CF, leading to defects in the heart’s electrical conduction system and HF (Lafuse et al. 2020). In this process, M2 macrophages are an important factor in the occurrence and development of CF (Kim et al. 2021). Therefore, a better understanding of the phenotype, function, and plasticity of macrophages during cardiac tissue damage and repair may lead to the development of new therapies to mitigate the progression of CF, thereby improving the prognosis of patients with MI (Lafuse et al. 2020). In this study, we provide evidence showing that enhancing macrophage glycogen metabolism can drive M2 macrophage differentiation into the M1 macrophages, thereby inhibiting fibroblast activation and indirectly inhibiting CF.

Macrophages are common cellular cells found in all tissues and compartments of the body under homeostatic physiological conditions (Gordon and Taylor 2005). In addition, macrophages acquire different phenotypes and exhibit various functions in the context of different diseases and in different stages of the same disease (Peet et al. 2020; Kaukonen et al. 2015; Bosmann and Ward 2013). In this study, we found that more macrophages had infiltrated human fibrotic cardiomyocyte tissue normal human heart tissue and that these macrophages largely exhibited an M2 phenotypes. We obtained similar results with MI model mice. Although macrophages in the CF context expressed high levels of inflammatory factors such as IL-6 to promote fibrosis, these macrophages also highly expressed anti-inflammatory factors such as TGF-β and IL-10, which have been shown in previous reports to be the main factors leading to CF (Revelo et al. 2021; Khalil et al. 2017; Su et al. 2017). This evidence suggests that M2 macrophages may play important roles in the development of CF. Targeting M2 macrophages in the CF microenvironment to drive their polarization into M1 macrophages and reduce the expression of profibrotic factors such as TGFβ while activating the expression of inflammatory factors may be an ideal treatment strategy for CF. In our previous studies, we showed that glycogen metabolism mediates the phenotype fate and function of macrophages and that macrophages synthesize glycogen in large quantities after glucose uptake to promote glycogen metabolism and activate the UDPG-P2Y_14_-STAT1 signaling pathway, which promotes inflammatory factor expression in macrophages (Ma et al. 2020). In this study, we preliminarily screened a glycogen metabolism activator, NaW, whose aqueous solution is weakly alkalinizing and is currently mainly used as a treatment for diabetes and anti-obesity in research studies (Amigó-Correig et al. 2011; Nocito et al. 2012; Hanzu et al. 2010; Barberà et al. 1994). In different animal models of type I and type II diabetes, NaW has been shown to normalize blood glucose levels without causing hypoglycemic episodes and is thus an effective and safe hypoglycemic agent (Muñoz et al. 2001; Barberà et al. 2001). NaW is also a phosphatase inhibitor that phosphorylates Ser9 in the regulatory subunit of GSK3β, reducing its catalytic activity and thereby reducing the phosphorylation rate of Gys1 (the inactive form). The increase in unphosphorylated Gys1 promotes glycogen synthesis and metabolism (Domínguez et al. 2003; Zafra et al. 2013). However, the findings from the present study provide evidence indicating that NaW activates glycogen metabolism in M2 macrophages and appropriately polarizes them to be M1 macrophages. Consistent with this finding, our RNA-seq results showed that NaW not only promoted the expression of inflammatory factors in macrophages but also promoted glucose uptake, thereby providing raw materials for glycogen synthesis. The mechanism by which NaW promotes glucose uptake by macrophages was also explored.

Another important finding in this study showed that NaW regulates lysosomal pH by increasing TMEM175 expression, which promotes glucose uptake in macrophages. Phagocytosis of extracellular substances and their effective degradation in lysosomes are the core functions of macrophages (Appelqvist et al. 2013). Lysosomes are membrane-bound spherical vesicular organelles that contain a variety of hydrolytic enzymes that breakdown a variety of biomolecules (Li et al. 2019; Kolter and Sandhoff 2005). Notably, this degradation process depends on the acidic pH of the lysosomal lumen. Previous reports have shown that the lysosome is an acidic reservoir of Ca^2+^ (Christensen et al. 2002; Morgan et al. 2011). NaW elevates the lysosomal pH and is a key factor that releases Ca^2+^ from these acidic reservoirs and promotes the translocation of TFEB, a cytoplasmic transcription factor. It is a key regulator of lysosomal biogenesis and M1-related glucose uptake and glycolysis (Medina et al. 2015; Roczniak-Ferguson, et al. 2012). This further increases the content of glucose, the raw material for glycogen synthesis, and promotes the flow of glycogen metabolism. However, how does NaW increase the pH of lysosomes?

Previous studies have shown that intracellular lysosomes require vacuolar H^+^ ATPase (V-ATPase) to establish a 50- to 5000-fold proton concentration gradient across the lysosome membrane to maintain a pH between 4.5 and 5.0, which is necessary for optimal activity of most hydrolytic enzymes in lysosomes (Mindell 2012). TMEM175 was previously considered a widely expressed K^+^-leaking channel protein in the lysosome membrane (Brunner 2020; Cang et al. 2015; Lee et al. 2017), but a recent study revealed that it is highly and selective permeable to protons and mediates lysosome H^+^ leakage; therefore, together with V-ATPase, TMEM175 maintains lysosomal pH homeostasis (Hu 2022). In this study, we found that NaW treatment promoted TMEM175 expression to increase lysosomal pH but exerted little effect on V-ATPase function. Lysosomes subsequently released Ca^2+^ and promoted TFEB nuclear translocation to increase glucose transporter expression, thereby promoting glucose influx into cells. Together, our findings reveal a mechanism through which NaW regulates lysosomal pH by promoting TMEM175 expression, thereby enhancing glycogen metabolism in macrophages. However, the mechanism by which NaW regulates TMEM175 function still needs to be explored in future work. In vitro, NaW indirectly inhibited fibroblast activation by inducing macrophage phenotype changes, and in vivo, NaW attenuated fibrosis progression and increased cardiac function in models of CF. Epidemiological studies have shown that premenopausal women have a lower incidence of cardiovascular disease than men of the same age because estrogen and related genetic and epigenetic modifications protect the cardiovascular system, giving them a large degree of protection from coronary artery disease, myocardial infarction, hypertension and pathological heart remodeling (e.g., cardiac fibrosis) (Kessler et al. 2019). In animal models, gene expression profiles also showed that male rat hearts, with stronger extracellular matrix-related gene induction and stronger mitochondrial gene inhibition, may be more susceptible to cardiac fibrosis than female rat hearts (Cavasin et al. 2004; Witt et al. 2008). Therefore, sex is a key influencing factor in the progression of cardiac fibrosis in both human and animal models, and estrogen may act as a protective factor, making women insensitive to pro-fibrosis influencing factors. In order to be more consistent with the actual situation of epidemiological research and ensure the success rate of scientific research on disease models, our primary focus was on investigating the male mouse cardiac fibrosis model in this study; however, we also conducted experiments involving female mice with cardiac fibrosis. It is also proved that NaW can regulate the polarization of M2-type macrophages to M1-type macrophages to improve cardiac fibrosis to some extent (Supplementary Fig. 6). In summary, these findings provide a new idea for anti-CF therapy and may lay the foundation for future clinical translational applications.

Antifibrosis therapy options are limited because fibrosis pathogenesis involves multiple factors, such as inflammatory responses and mitochondrial dysfunction. Different cell types contribute differently to fibrosis progression, and cross-linking among cells is common. Despite the fact that M2 macrophages secrete TGFβ, a pivotal cytokine in the fibrotic process, there are still studies demonstrating that macrophages can influence fibroblasts through secretion of other cellular factors (Hesketh et al. 2017). Recent investigations have revealed that macrophage connexin channels can mediate ATP exocytosis, inducing a cytosolic calcium response in neighboring fibroblasts, thereby promoting their activation and facilitating fibrosis formation (Bhattacharyya 2022). Simultaneously, macrophage-derived exosomes can encapsulate microRNAs to establish interaction with fibroblasts (Kishore and Petrek 2021). Furthermore, by secreting oncostatin M to alleviate ischemic injury and promote repair processes, macrophages are capable of stimulating fibroblast activation (Song 2023). Collectively, these studies underscore the role of macrophages as signal transmitters engaging in paracrine mechanisms to regulate the fibrotic response. The role of microRNAs and lymphangiogenesis as crucial regulators of the inflammatory response and fibrosis progression necessitates further investigation to determine their therapeutic efficacy (Marracino et al. 2021; Varzideh et al. 2022; Heron et al. 2023). In clinical trials studies reported thus far, the effects of anti-fibrosis agents have been disappointing. Promising data have mainly been reported for treatment with renin–angiotensin–aldosterone system (RAAS) inhibitors (Fang et al. 2017). However, the effectiveness of these drugs in clinical studies has been nominal, showing only mild regression of CF, which persisted in patients with HF even after they received the accepted standard treatment (Fang et al. 2017; Querejeta et al. 2004). Some new strategies to mediate the direct attenuation of fibrosis, such as engineered chimeric T-cell antigen receptor (CAR-T) therapy, have been used to target muscle fibroblasts and thus eliminate fibrosis, but because of fibroblast perseverance, we still need to find more muscle fibroblast-specific tags and perform more experiments to ensure drug safety (Aghajanian et al. 2019; Feins et al. 2019). Our study provides a new antifibrotic strategy and lays a foundation for the clinical application of glycogen metabolism regulation to induce macrophage phenotype switching. In addition, we reveal the regulatory mechanism underlying the effect of the small-molecule compound NaW on macrophage glycogen metabolism and lysosomes. Notably, NaW may show the following advantages as an antifibrosis drug: (1) NaW is a small compound that readily distributes throughout the fibrosis microenvironment and targets M2 macrophages; (2) NaW does not eliminate M2 macrophages but reprograms them to acquire the M1 phenotype, which can be better utilized to reconstruct the cardiac immune microenvironment; and (3) NaW is a very safe drug that has been tested in clinical trials.

The heart, being a highly metabolically active organ, possesses the inherent characteristic of adaptability. In fact, it has the ability to efficiently switch between various substrates in order to fulfill its energy requirements while optimizing nutrient availability. During diabetes-induced diastolic decline and cardiac fibrosis, the metabolic flexibility is disrupted, leading to a significant decrease in glycolysis contribution to cardiac ATP production and an increased reliance on fatty acid oxidation (Jankauskas et al. 2021). Patients treated with the sodium-glucose cotransporter 2 inhibitor (SGLT2i) dapagliflozin have reported improvements in diastolic function and cardiac fibrosis (Lee et al. 2022). It is important to note that the heart’s capacity to store energy substrates such as glycogen and triglycerides is limited, therefore enhancing cardiac function relies on its ability to increase glycogen synthesis or fatty acid utilization (Lopaschuk et al. 2021; Opie et al. 1971). NaW, as a glycogen metabolism activator, may enhance cardiac function by promoting glycogen synthesis and alleviating diabetes-induced decline in cardiac function. Additionally, MI refers to myocardial damage caused by acute ischemia and hypoxia of the heart accompanied by extensive cardiomyocyte apoptosis. The excessive production of reactive oxygen species further exacerbates cardiomyocyte damage and apoptosis. The AHR agonist ITE mitigates cardiomyocyte apoptosis by activating the Akt/p70S6K signaling pathway, thereby attenuating left ventricular remodeling and improving cardiac dysfunction following myocardial infarction (Lin et al. 2023). Our findings demonstrate that NaW enhances glucose uptake by cardiomyocytes and activates the pentose phosphate pathway (PPP) for NADPH production (Supplementary Fig. 7A–C). Activation of this process may promote cardiomyocyte survival during MI; however, further experiments are required for validation.

In summary, the data from this study clearly show that the M2 macrophages in the context of CF is reprogramed to acquire an M1 phenotype by increasing macrophage glycogen metabolism and that TMEM175 mediates NaW regulatory effects on macrophage phenotype and metabolism by changing lysosomal pH. The results suggests that NaW is a true immunomodulator that exerts an anti-CF effect. Together, these findings may open up a new avenue for CF immunotherapy.

### Supplementary Information

Supplementary Material 1. Supplementary Fig. 1 **(A)** Wilcoxon tests were performed to show the proportions of various types of immune cells that infiltrated in tissues from patients with and without heart failure (HF). **(B)** The distribution of each cluster of infiltrating macrophages in tissues from patients with and without HF is shown in the T-distributed stochastic neighbor embedding (t-SNE) projection. (0: ACSM3^+^ macrophages; 1: APOD^+^ macrophages; 2: FBN1^+^ macrophages; 3: FMOD^+^ macrophages; 4: IL1R2^+^macrophages; 5: LDB3^+^ macrophages; 6: MRC1^+^ macrophages; 7: NEGR1^+^ macrophages; 8: NPPB^+^ macrophages; 9: SPHKAP^+^ macrophages; and 10: TECRL^+^ macrophages). **(C)** KEGG results are presented in a bar plot.

Supplementary Material 2. Supplementary Fig. 2. The bone marrow-derived macrophages (BMDMs) cultured under IL-4 conditions were incubated with or without NaW for 12 hours, followed by the subsequent withdrawal of NaW. (**A**-**D**) The expression levels of Arg1, Tgfβ1, Il10, Tnf, Il6, and Nos2 in M2 macrophages were analyzed using real-time PCR before and after treatment with NaW. Additionally, the expression levels were also measured at 24 hours and 48 hours after withdrawal of NaW (**A**). IL-10, TGFβ, TNF, IL-6, iNOS and Arg-1 levels were measured by ELISAs (**B**-**C**) and western blotting (**D**). *G6pdx* and *6Pdg* expression levels in fibroblasts was measured by real-time PCR (**E**) and western blotting (**F**). (**G**) Reactive oxygen species (ROS) levels and mean fluorescence intensity (MFI) of the LysoSensor probe were measured by flow cytometry. The data are presented as the mean ± SEM. P values were calculated by one-way ANOVA. *P < 0.05; **P < 0.01; ***P < 0.001.

Supplementary Material 3. Supplementary Fig. 3 (**A**, **B**) GO results are represented in directed acyclic graphs (**A**) and bubble plots (**B**). (**C**) Differentially expressed genes (DEGs) are shown in the heatmap.

Supplementary Material 4. Supplementary Fig. 4 (**A**) *Slc2a2*, *Slc2a4* and *Slc2a8* expression in BMDMs was measured by real-time PCR. (**B**) *V0c*, *Voe*, *V1a*, *V1b2*, *V1c1* and *V1e1* expression in BMDMs was measured by real-time PCR. (C, D) BMDMs were transfected with *Tmem175* siRNA (**C**) and plasmid (**D**) and stimulated with IL-4 for 24 hours. *Tmem175* expression was measured by real-time PCR. (**E**) *Tpcn1* and *Tpcn2* expression in BMDMs was measured by real-time PCR. (**F**) BMDMs were transfected with Tfeb siRNA and stimulated with IL-4 for 24 hours, and *Tfeb* expression was measured by real-time PCR. Unless otherwise specified, n = 3 biologically independent experiments. The data are presented as the mean ± SEM. P values were calculated by one-way ANOVA. *P < 0.05; **P < 0.01; ***P < 0.001.

Supplementary Material 5. Supplementary Fig. 5 Thirty-eight days after the sham/MI operation, the hearts of sham, sham-NaW, MI-PBS and MI-NaW mice were observed by immunofluorescence. (**A**) Representative immunofluorescence (left) and statistical analysis (right) of F4/80+ macrophages within the fibrotic region. The M2 macrophages isolated from the hearts of two groups of mice (3MI-PBS vs. 3MI-NaW) were subjected to RNA-seq. (**B**) GO results are represented using bubble plots. (**C**) KEGG results are presented in a bar plot. (**D**) DEGs in a volcano plot. (**E**) The expression of *Pygl* in macrophages was measured by real-time PCR. The data are presented as the mean ± SEM. P values were calculated by one-way ANOVA. *P < 0.05; **P < 0.01; ***P < 0.001.

Supplementary Material 6. Supplementary Fig. 6 (**A**-**B**) Thirty-eight days after the sham/MI operation, the hearts of sham, sham-NaW, MI-PBS and MI-NaW mice were observed by Masson’s trichrome staining and microscopy (scale bar, 1 mm) (**A**), and the fibrotic region areas were quantified (**B**), n=6. (**C**) Representative immunostaining (scale bar, 10 μm) for α-SMA in fibrotic regions of the heart 38 days after the sham/MI operation. (**C**) Blue: hematoxylin; brown: a-SMA. (**D**, **E**) Representative images showing collagen I (**D**) and III (**E**) immunofluorescence staining. Scale bar, 20 μm. (**F**-**H**) Ejection fraction (EF) (F), end systolic volume (ESV) (**G**) and end-diastolic volume (EDV) (**H**) as quantified via echocardiography 38 days after the sham/MI operation; n=9, 9, 9, and 9, respectively. (**I**-**M**) On the 38th day after MI surgery, F4/80+ macrophages were isolated from the fibrotic area of MI-PBS, MI-NaW, sham, and sham-NaW mice, and the expression of *Tgfb1*, *Mrc1*, *Arg1*, *Nos2* and *Tnf* in macrophages was measured by real-time PCR; n=8, 8, 8, and 8, respectively. The data are presented as the mean ± SEM. P values were calculated by one-way ANOVA. *P < 0.05; **P < 0.01; ***P < 0.001.

Supplementary Material 7. Supplementary Fig. 7 The neonatal mouse cardiomyocytes were isolated and subjected to a 24h treatment with NaW. (**A**) Fluorescence intensity analysis of cardiomyocytes after 2-NBDG administration for 30 min. The expression of *Slc2a4*, *G6pdx*, *6Pdg* (**B**) and NADPH/NADP+ (**C**) in cardiomyocytes with or without NaW treatment was analyzed. The data are presented as the mean ± SEM. P values were calculated by one-way ANOVA. *P < 0.05; **P < 0.01; ***P < 0.001.

Supplementary Material 8.

## Data Availability

The authors confirm that the data supporting the findings of this study are available within the article and its Additional materials.
