# Supplementary figures and images for: Modulation of anti-cardiac fibrosis immune responses by changing M2 macrophages into M1 macrophages
Source: Mol Med. 2024 Jun 15;30:88. doi: 10.1186/s10020-024-00858-z (PMC11179216; doi:10.1186/s10020-024-00858-z)

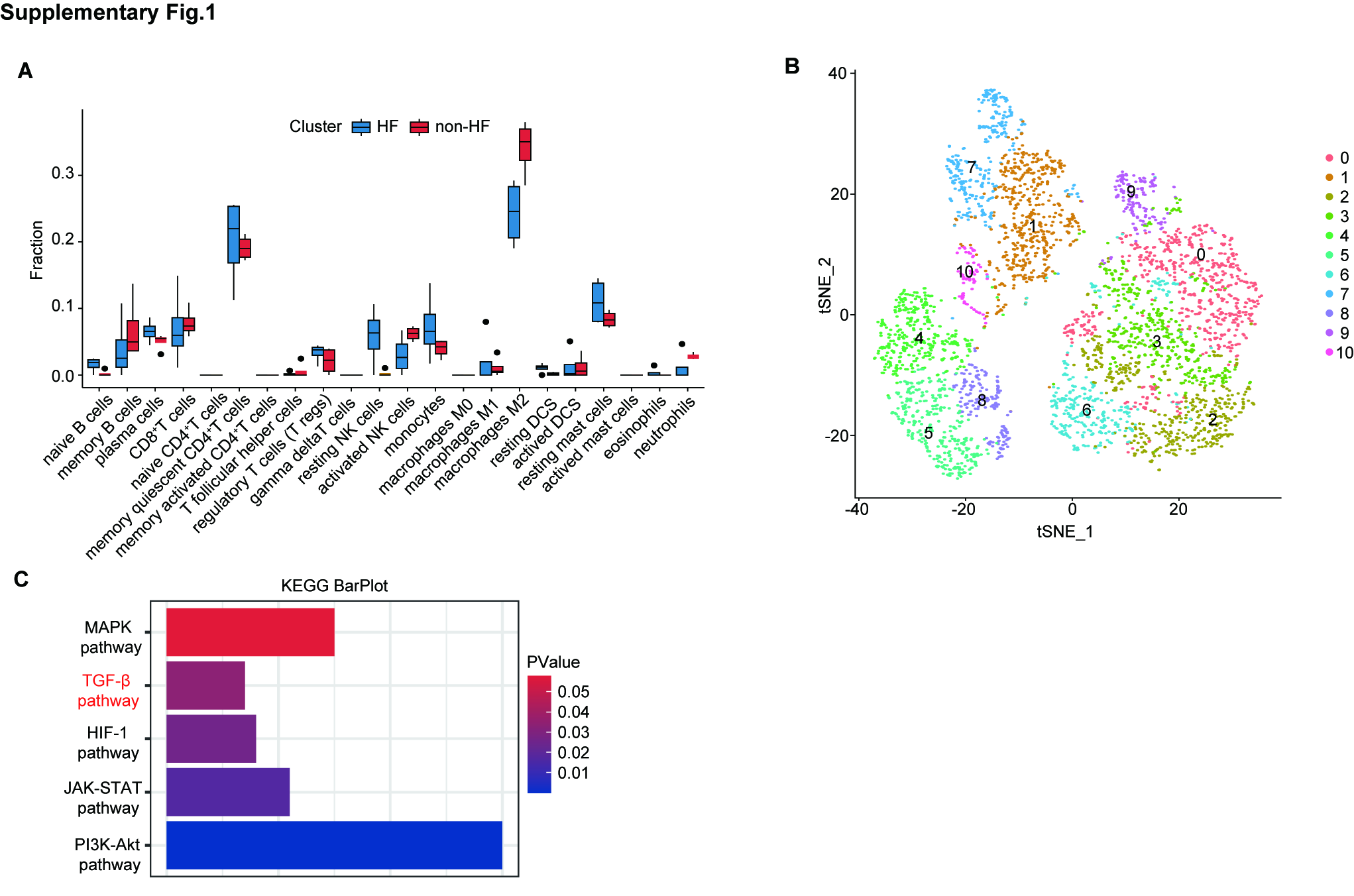

Supplement: Supplementary file 1 — Supplementary Material 1. Supplementary Fig. 1 (A) Wilcoxon tests were performed to show the proportions of various types of immune cells that infiltrated in tissues from patients with and without heart failure (HF). (B) The distribution of each cluster of infiltrating macrophages in tissues from patients with and without HF is shown in the T-distributed stochastic neighbor embedding (t-SNE) projection. (0: ACSM3+ macrophages; 1: APOD+ macrophages; 2: FBN1+ macrophages; 3: FMOD+ macrophages; 4: IL1R2+macrophages; 5: LDB3+ macrophages; 6: MRC1+ macrophages; 7: NEGR1+ macrophages; 8: NPPB+ macrophages; 9: SPHKAP+ macrophages; and 10: TECRL+ macrophages). (C) KEGG results are presented in a bar plot. [file 10020_2024_858_MOESM1_ESM.tif]

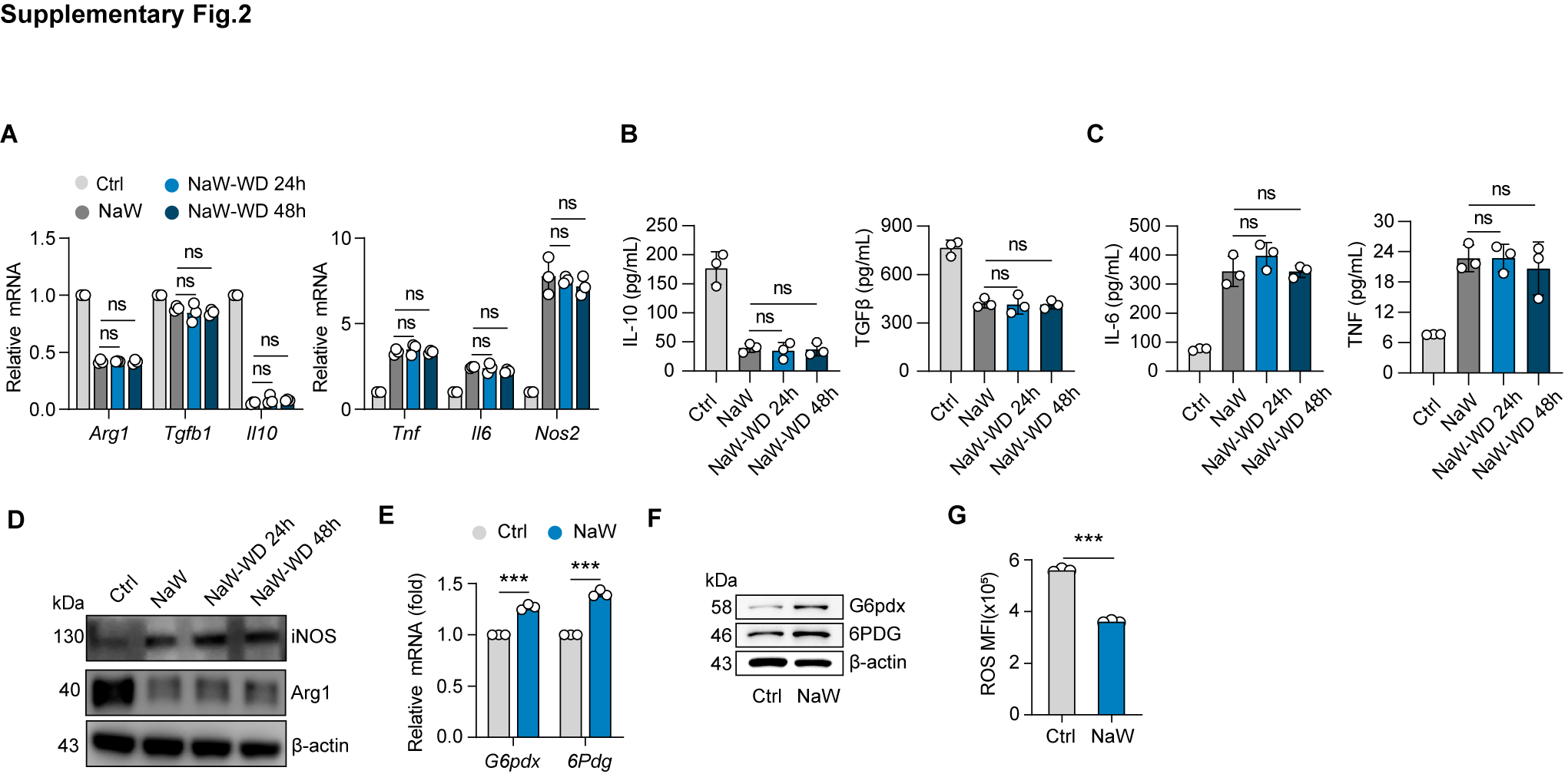

Supplement: Supplementary file 2 — Supplementary Material 2. Supplementary Fig. 2. The bone marrow-derived macrophages (BMDMs) cultured under IL-4 conditions were incubated with or without NaW for 12 hours, followed by the subsequent withdrawal of NaW. (A-D) The expression levels of Arg1, Tgfβ1, Il10, Tnf, Il6, and Nos2 in M2 macrophages were analyzed using real-time PCR before and after treatment with NaW. Additionally, the expression levels were also measured at 24 hours and 48 hours after withdrawal of NaW (A). IL-10, TGFβ, TNF, IL-6, iNOS and Arg-1 levels were measured by ELISAs (B-C) and western blotting (D). G6pdx and 6Pdg expression levels in fibroblasts was measured by real-time PCR (E) and western blotting (F). (G) Reactive oxygen species (ROS) levels and mean fluorescence intensity (MFI) of the LysoSensor probe were measured by flow cytometry. The data are presented as the mean ± SEM. P values were calculated by one-way ANOVA. *P < 0.05; **P < 0.01; ***P < 0.001. [file 10020_2024_858_MOESM2_ESM.tif]

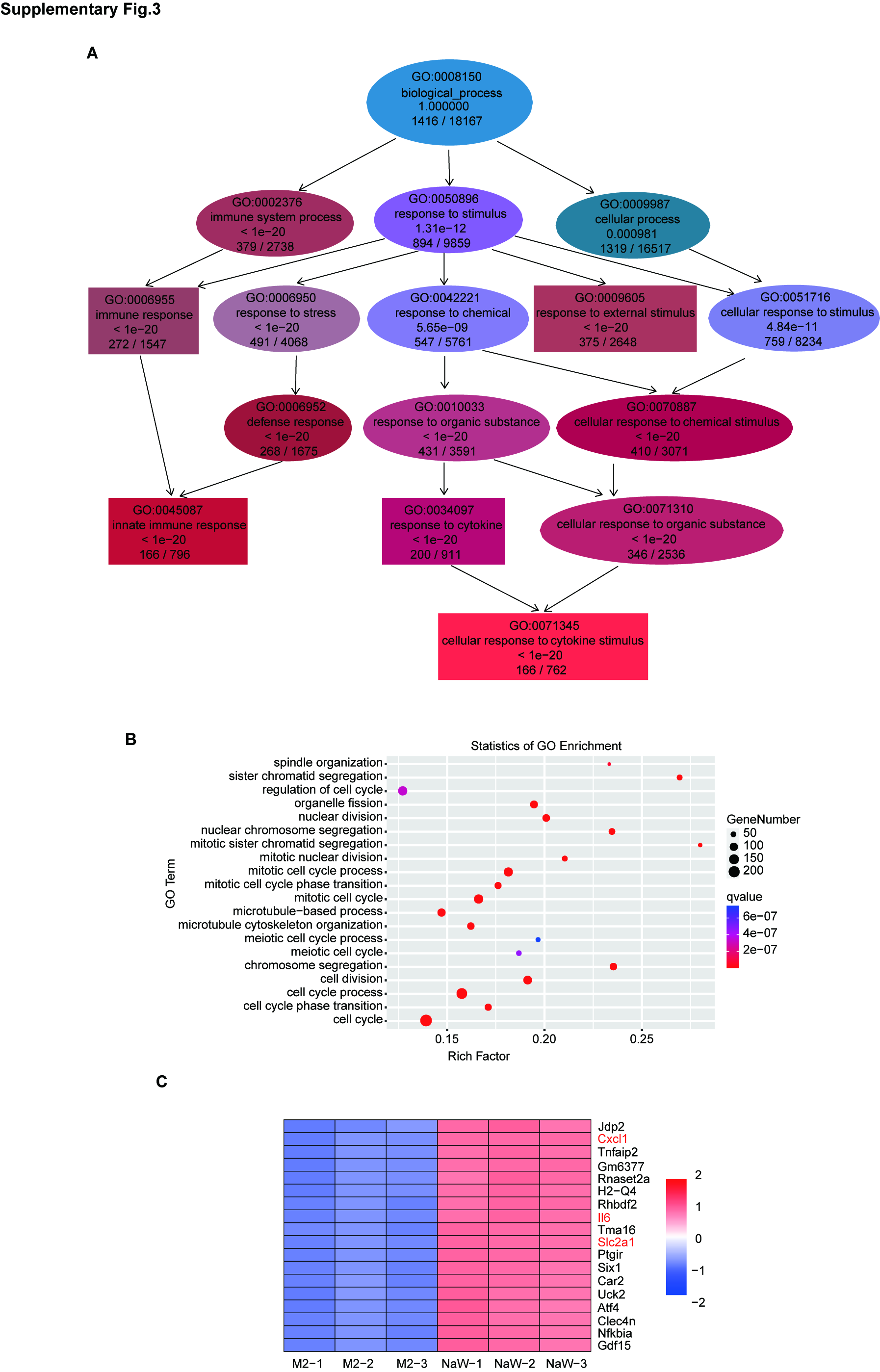

Supplement: Supplementary file 3 — Supplementary Material 3. Supplementary Fig. 3 (A, B) GO results are represented in directed acyclic graphs (A) and bubble plots (B). (C) Differentially expressed genes (DEGs) are shown in the heatmap. [file 10020_2024_858_MOESM3_ESM.tif]

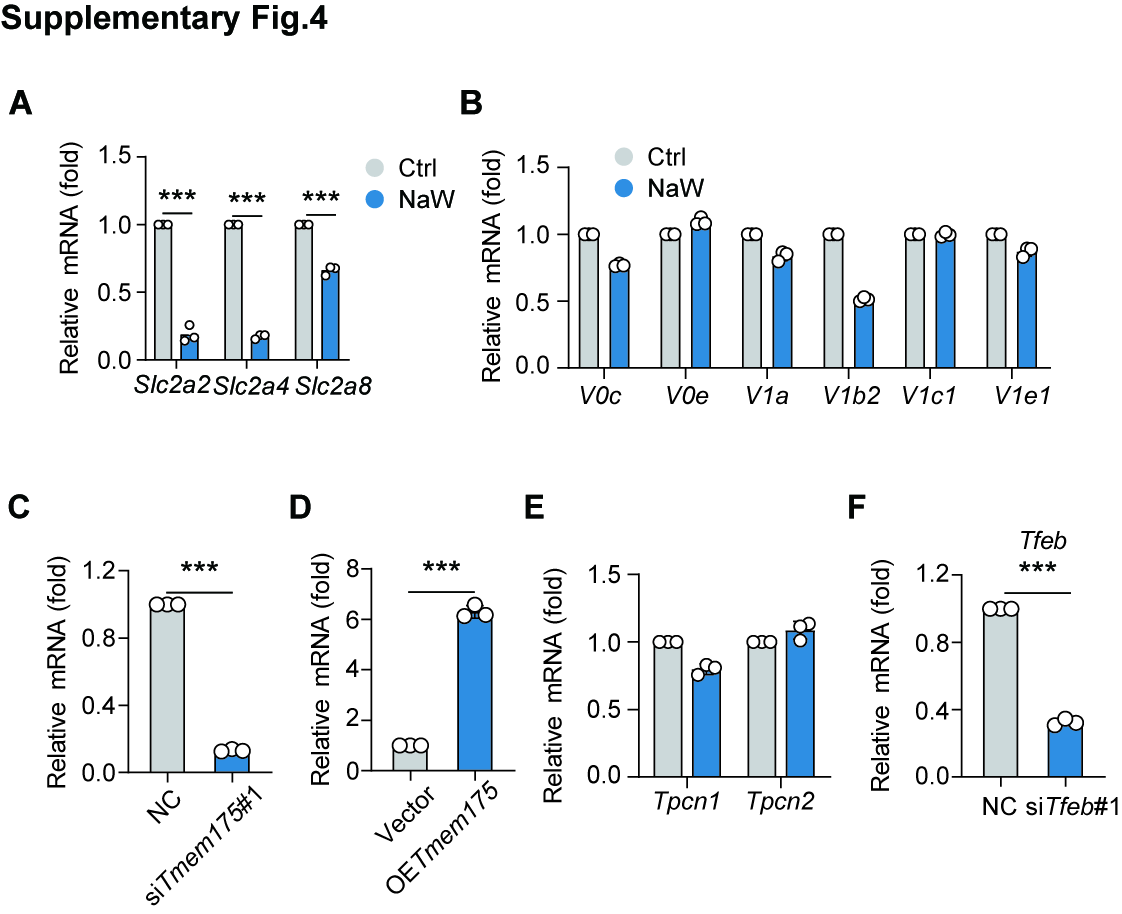

Supplement: Supplementary file 4 — Supplementary Material 4. Supplementary Fig. 4 (A) Slc2a2, Slc2a4 and Slc2a8 expression in BMDMs was measured by real-time PCR. (B) V0c, Voe, V1a, V1b2, V1c1 and V1e1 expression in BMDMs was measured by real-time PCR. (C, D) BMDMs were transfected with Tmem175 siRNA (C) and plasmid (D) and stimulated with IL-4 for 24 hours. Tmem175 expression was measured by real-time PCR. (E) Tpcn1 and Tpcn2 expression in BMDMs was measured by real-time PCR. (F) BMDMs were transfected with Tfeb siRNA and stimulated with IL-4 for 24 hours, and Tfeb expression was measured by real-time PCR. Unless otherwise specified, n = 3 biologically independent experiments. The data are presented as the mean ± SEM. P values were calculated by one-way ANOVA. *P < 0.05; **P < 0.01; ***P < 0.001. [file 10020_2024_858_MOESM4_ESM.tif]

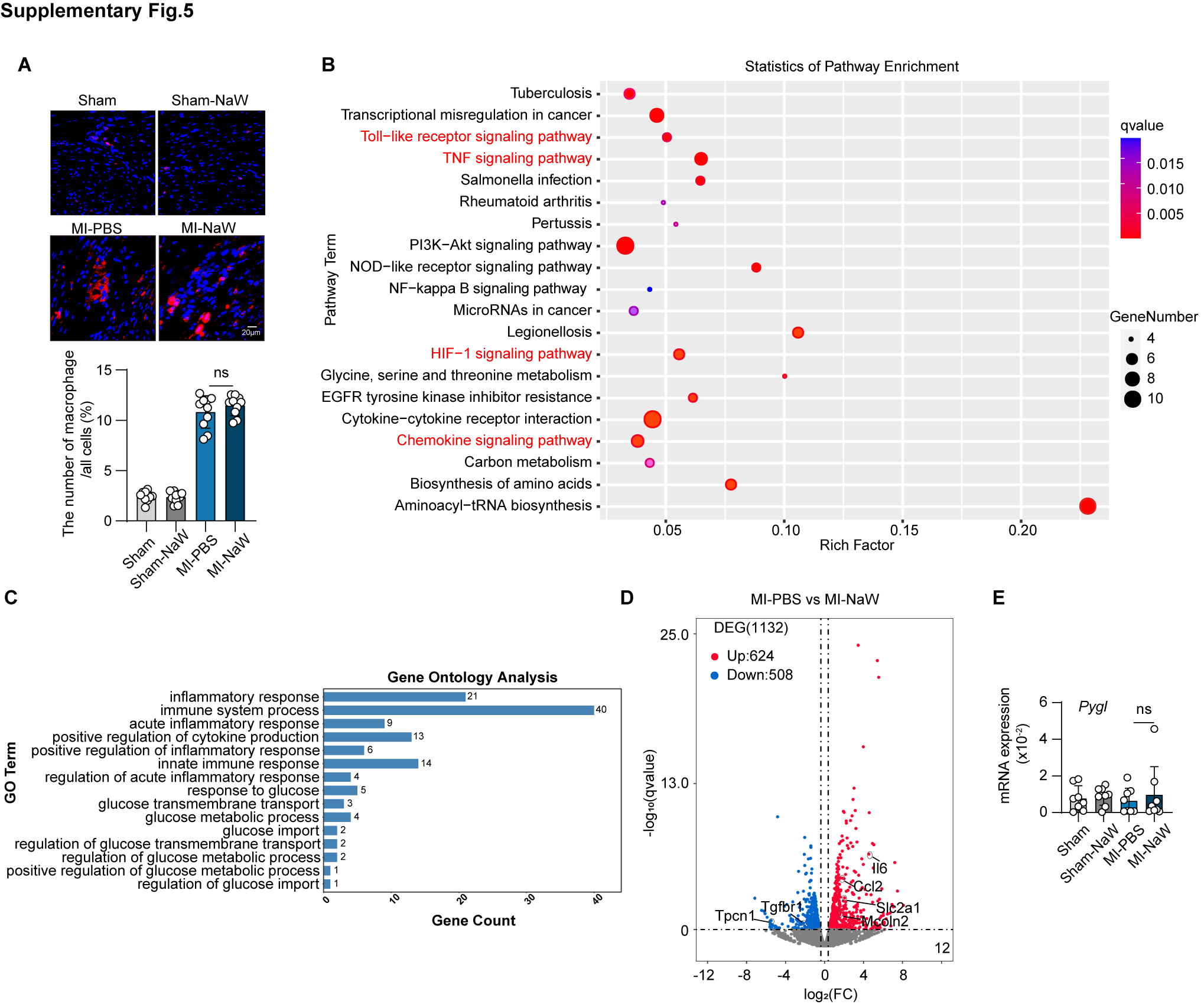

Supplement: Supplementary file 5 — Supplementary Material 5. Supplementary Fig. 5 Thirty-eight days after the sham/MI operation, the hearts of sham, sham-NaW, MI-PBS and MI-NaW mice were observed by immunofluorescence. (A) Representative immunofluorescence (left) and statistical analysis (right) of F4/80+ macrophages within the fibrotic region. The M2 macrophages isolated from the hearts of two groups of mice (3MI-PBS vs. 3MI-NaW) were subjected to RNA-seq. (B) GO results are represented using bubble plots. (C) KEGG results are presented in a bar plot. (D) DEGs in a volcano plot. (E) The expression of Pygl in macrophages was measured by real-time PCR. The data are presented as the mean ± SEM. P values were calculated by one-way ANOVA. *P < 0.05; **P < 0.01; ***P < 0.001. [file 10020_2024_858_MOESM5_ESM.tif]

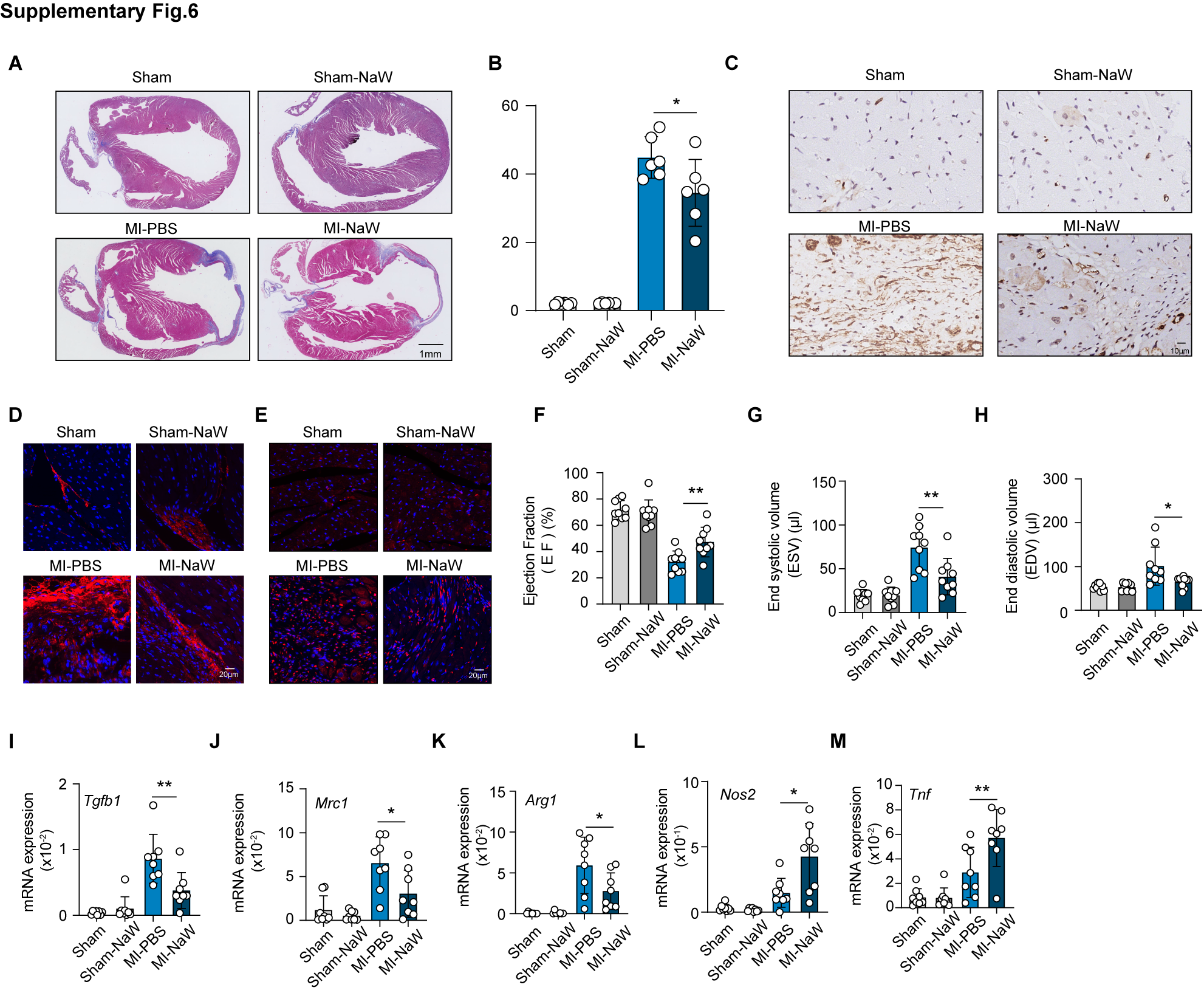

Supplement: Supplementary file 6 — Supplementary Material 6. Supplementary Fig. 6 (A-B) Thirty-eight days after the sham/MI operation, the hearts of sham, sham-NaW, MI-PBS and MI-NaW mice were observed by Masson’s trichrome staining and microscopy (scale bar, 1 mm) (A), and the fibrotic region areas were quantified (B), n=6. (C) Representative immunostaining (scale bar, 10 μm) for α-SMA in fibrotic regions of the heart 38 days after the sham/MI operation. (C) Blue: hematoxylin; brown: a-SMA. (D, E) Representative images showing collagen I (D) and III (E) immunofluorescence staining. Scale bar, 20 μm. (F-H) Ejection fraction (EF) (F), end systolic volume (ESV) (G) and end-diastolic volume (EDV) (H) as quantified via echocardiography 38 days after the sham/MI operation; n=9, 9, 9, and 9, respectively. (I-M) On the 38th day after MI surgery, F4/80+ macrophages were isolated from the fibrotic area of MI-PBS, MI-NaW, sham, and sham-NaW mice, and the expression of Tgfb1, Mrc1, Arg1, Nos2 and Tnf in macrophages was measured by real-time PCR; n=8, 8, 8, and 8, respectively. The data are presented as the mean ± SEM. P values were calculated by one-way ANOVA. *P < 0.05; **P < 0.01; ***P < 0.001. [file 10020_2024_858_MOESM6_ESM.tif]

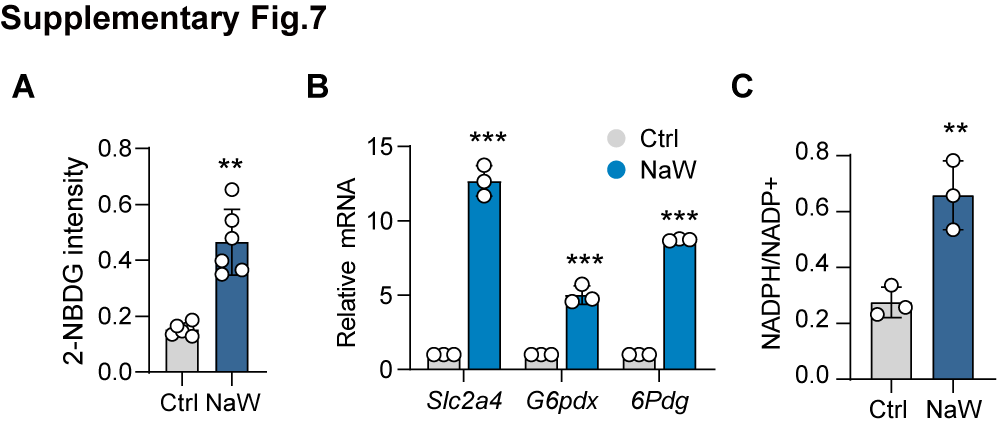

Supplement: Supplementary file 7 — Supplementary Material 7. Supplementary Fig. 7 The neonatal mouse cardiomyocytes were isolated and subjected to a 24h treatment with NaW. (A) Fluorescence intensity analysis of cardiomyocytes after 2-NBDG administration for 30 min. The expression of Slc2a4, G6pdx, 6Pdg (B) and NADPH/NADP+ (C) in cardiomyocytes with or without NaW treatment was analyzed. The data are presented as the mean ± SEM. P values were calculated by one-way ANOVA. *P < 0.05; **P < 0.01; ***P < 0.001. [file 10020_2024_858_MOESM7_ESM.tif]
