# Supplementary material for: Modulation of anti-cardiac fibrosis immune responses by changing M2 macrophages into M1 macrophages
Source: Mol Med. 2024 Jun 15;30:88. doi: 10.1186/s10020-024-00858-z (PMC11179216; doi:10.1186/s10020-024-00858-z)
Supplement: Supplementary file 8 — Supplementary Material 8. [file 10020_2024_858_MOESM8_ESM.docx]

**Supplementary Table 1. Primer sequence.**

| **GENE** | **PRIMER** | **PRIMER SEQUENCE** |
| --- | --- | --- |
| Mouse *Nos2* | FW | GAGACAGGGAAGTCTGAAGCAC |
|  | RV | CCAGCAGTAGTTGCTCCTCTTC |
| Mouse *Tnf* | FW | GGTGCCTATGTCTCAGCCTCTT |
|  | RV | GCCATAGAACTGATGAGAGGGAG |
| Mouse *Il6* | FW | TAGTCCTTCCTACCCCAATTTCC |
|  | RV | TTGGTCCTTAGCCACTCCTTC |
| Mouse *Il10* | FW | CGGGAAGACAATAACTGCACCC |
|  | RV | CGGTTAGCAGTATGTTGTCCAGC |
| Mouse *Arg1* | FW | CATTGGCTTGCGAGACGTAGAC |
|  | RV | GCTGAAGGTCTCTTCCATCACC |
| Mouse *Mrc1* | FW | GTTCACCTGGAGTGATGGTTCTC |
|  | RV | AGGACATGCCAGGGTCACCTTT |
| Mouse *Tfeb* | FW | CGCCTGGAGATGACTAACAAGC |
|  | RV | GGCAACTCTTGCTTCACCACCT |
| Mouse *Pygl* | FW | GGCAGAAGTGGTGAACAATGACC |
|  | RV | TCCGATAGGTCTGTGGCTGGAA |
| Mouse *Ugp2* | FW | CTGATGAACCCACCCAATGGGA |
|  | RV | GAGCGATTTCCACCAGTCTCAG |
| Mouse *P2Y_14_* | FW | ACCTCCGTCAAGAGGAAGTCCA |
|  | RV | GCTGTAGTGACCTTCCGTCTGA |
| Mouse *6Pgd* | FW | CATCGCTGCAAAAGTGGGAACC RV AGCCTCACAGATGAGCTGCATG |
|  | RV | AGCCTCACAGATGAGCTGCATG |
| Mouse *G6pdx* | FW | GACCAAGAAGCCTGGCATGTTC |
|  | RV | AGACATCCAGGATGAGGCGTTC |
| Mouse *Tmem175* | FW | ATCAGCAAGGCTACCACCTGGT |
|  | RV | GGCATAGACACCGTCGCTGAAA |
| Mouse *Pygl* | FW | GGCAGAAGTGGTGAACAATGACC |
|  | RV | TCCGATAGGTCTGTGGCTGGAA |
| Mouse *Slc2a1* | FW | CAGTTCGGCTATAACACTGGTG |
|  | RV | GCCCCCGACAGAGAAGATG |
| Mouse *Atp6v0c* | FW | GACTGATGGCATCACCCTCTAC |
|  | RV | CAGGATCATGCCCACGAACAGT |
| Mouse *Atp6v0e* | FW | CTCCTCGTGCCCTGGTTTATCC |
|  | RV | GTGGTCCAAACAGAGGATTGAGC |
| Mouse *Atp6v1a* | FW | GCTGGCTTCTTTCTATGAGCGAG |
|  | RV | GCGTTGCAGAAGTGACTGGATC |
| Mouse *Atp6v1b2* | FW | ATGCGGGGAATCGTGAACG |
|  | RV | AGGCTGGGATAGGTAGTTCCG |
| Mouse *Atp6v1c1* | FW | GGTTGGCTTGTCGGATGAACTG |
|  | RV | TGTCCTCCAGCACATCAGCCAT |
| Mouse *Atp6v1e1* | FW | AAAGGTCGCCTTGTGCAAACGC |
|  | RV | CTCTGAGGACTTTGAGCCTTGC |
| Mouse *Mcoln1* | FW | ACCATCTCGGGGACTGTCAT |
|  | RV | CAGGTAGCGAATGACACCGA |
| Mouse *Mcoln2* | FW | GCATTCTGGTGTGGCTGTTC |
|  | RV | GGTGTGGTAAGAGTCGGTG |
| Mouse *Tpcn1* | FW | CCCTGGAGTTACCTCGTGTTTC |
|  | RV | GAATGCCGTGACCGAGAAATCG |
| Mouse *Tpcn2* | FW | CATCCACCTGTGTCTCTTCACC |
|  | RV | GTGAGGTCAGTGCTTCTGGAAG |
| Mouse *Actb* | FW | GGCTGTATTCCCCTCCATCG |
|  | RV | CCAGTTGGTAACAATGCCATGT |

**Supplementary Table 2. siRNA Sequence.**

| Mouse *Pygl* | siRNA#1 | CCATTTACCAGCTTGGATT |
| --- | --- | --- |
| Mouse *Tmem175* | siRNA#1 | GGACTACAGCCCTGCTGCA |
| Mouse *Tfeb* | siRNA#1 | GCAGGCTGTCATGCATTAT |
